# Allantoin accumulation through overexpression of *ureide permease1* improves rice growth under limited nitrogen conditions

**DOI:** 10.1111/pbi.13054

**Published:** 2019-02-04

**Authors:** Mark Christian Felipe R. Redillas, Seung Woon Bang, Dong‐Keun Lee, Youn Shic Kim, Harin Jung, Pil Joong Chung, Joo‐Won Suh, Ju‐Kon Kim

**Affiliations:** ^1^ Graduate School of International Agricultural Technology and Crop Biotechnology Institute/GreenBio Science and Technology Seoul National University Pyeongchang Korea; ^2^ Center for Nutraceutical and Pharmaceutical Materials Division of Bioscience and Bioinformatics Myongji University Yongin Gyeonggi Korea; ^3^ Present address: Department of Biology De La Salle University Manila Philippines; ^4^ Present address: NUS Synthetic Biology for Clinical and Technological Innovation (SynCTI) Department of Biochemistry Yong Loo Lin School of Medicine National University of Singapore Singapore Singapore; ^5^ Present address: Temasek Life Science Laboratory National University of Singapore Singapore Singapore

**Keywords:** allantoin, amino acid, ammonium, nitrogen, *Oryza sativa*, rice, ureides

## Abstract

In legumes, nitrogen (N) can be stored as ureide allantoin and transported by ureide permease (UPS) from nodules to leaves where it is catabolized to release ammonium and assimilation to amino acids. In non‐leguminous plants especially rice, information on its roles in N metabolism is scarce. Here, we show that OsUPS1 is localized in plasma membranes and are highly expressed in vascular tissues of rice. We further evaluated an activation tagging rice overexpressing *OsUPS1* (*OsUPS1* ^*OX*^ ) under several N regimes. Under normal field conditions, panicles from *OsUPS1* ^*OX*^ plants (14 days after flowering (DAF)) showed significant allantoin accumulation. Under hydroponic system at the vegetative stage, plants were exposed to N‐starvation and measured the ammonium in roots after resupplying with ammonium sulphate. *OsUPS1* ^*OX*^ plants displayed higher ammonium uptake in roots compared to wild type (WT). When grown under low‐N soil supplemented with different N‐concentrations, *OsUPS1* ^*OX*^ exhibited better growth at 50% N showing higher chlorophyll, tiller number and at least 20% increase in shoot and root biomass relative to WT. To further confirm the effects of regulating the expression of *OsUPS1*, we evaluated whole‐body‐overexpressing plants driven by the *GOS2* promoter (*OsUPS1* ^*GOS*^ ^*2*^) as well as silencing plants (*OsUPS1* ^*RNA*^ ^*i*^). We found significant accumulation of allantoin in leaves, stems and roots of *OsUPS1* ^*GOS*^ ^*2*^ while in *OsUPS1* ^*RNA*^ ^*i*^ allantoin was significantly accumulated in roots. We propose that OsUPS1 is responsible for allantoin partitioning in rice and its overexpression can support plant growth through accumulation of allantoin in sink tissues which can be utilized when N is limiting.

## Introduction

In crop production, nitrogen (N) supply and availability greatly influence the growth and yield of plants. To support the increasing demand in crops due to the rapid growth of world population, it is essential for crop researchers and breeders to come up with strategies to sustain production and meet demands. Improvement in crop management and agronomy coupled with conventional breeding and genetic engineering have been the major factors behind increased crop production (Han *et al*., 2015). However, for several decades synthetic N in the form of ammonium fertilizers showed to be the most practical way to increase crop production. In global cereal production alone, excessive use of synthetic N showed a dramatic increase from 11.6 Tg in 1961 to 104 Tg in 2006 (Mulvaney *et al*., 2009). Though high N input can increase yield, plants however do not take up these N completely and more than 50% are leached in soils resulting in the contamination of the environment (Gruber and Galloway, 2008).

Nitrogen is transported in a plant vascular system in different forms depending on the plant species. Rice typically utilizes ammonia for assimilation to synthesize protein and other biochemical compounds once it enters the GS/GOGAT cycle (Xu *et al*., 2012). Other N forms such nitrate as well as asparagine and glutamine (amides) are also used for transport (Pate *et al*., 1980). Exogenous N is incorporated in plants in several steps starting from uptake followed by assimilation, translocation and recycling and remobilization at later stages each with its own enzymes and substrates. On the other hand, a number of N_2_‐fixing plants, which are capable of utilizing atmospheric N through symbiotic relationship with rhizobia found within nodules of the roots, transport N in a different form.

Tropical nodulating legumes utilize the ureides, allantoin and allantoic acid, as the main transport form of organic N (Alamillo *et al*., 2010; Desimone *et al*., 2002; Pate *et al*., 1980; Pelissier *et al*., 2004). When symbiotic bacteria are present in nodules of N_2_‐fixing plants, they produce ammonium which is then used for the synthesis of purines and uric acid. These uric acid are transported to neighbouring uninfected cells which are then used to synthesize allantoin in peroxisomes (Hanks *et al*., 1981). Allantoin and its first derivative allantoic acid play important roles in the assimilation and metabolism of N. In soybean, allantoin in cotyledons is transported to the shoot axis during germination (Duran and Todd, 2012) and concentration is highest in stems of green pods (Matsumoto *et al*., 1977). For example, tropical and sub‐tropical legumes such as *Vigna unguiculata* (cowpea), *Glycine max* (soybean) and *Phaseolus vulgaris* (French bean) dominantly transport ureides instead of amino acids under N‐fixing conditions. Ureides also amount to 20 or 10 mm in the stem tissue or xylem (Layzell and Larue, 1982; Rainbird *et al*., 1984) and 94 mm in nodule exudates (Pelissier *et al*., 2004; Streeter, 1979). These ureides are loaded into the network of nodule‐root xylem vessels and transported to aerial organs by the transpirational water current which is then enzymatically catabolized in series of reactions to yield ammonium (Baral *et al*., 2016). This ureide strategy is said to be more efficient than the amide strategy because of its lower carbon to N ratio, able to release four molecules of ammonium per allantoin, and utilized for plant survival under stressed conditions (Sagi *et al*., 1998; Todd *et al*., 2006; Watanabe *et al*., 2014). Allantoin level also responds to exogenous N status such that the concentration decreases under non‐nodulating conditions where the amount of N present in soil is high (Amarante *et al*., 2006; Pate *et al*., 1980).

Ureides are produced through purine degradation and oxidation in the purine metabolism pathway (Brychkova *et al*., 2008; Werner and Witte, 2011; Werner *et al*., 2010; Zrenner *et al*., 2006). Catabolism of allantoin involves the first key enzyme called allantoinase (ALN) which produces allantoate/allantoic acid (Werner *et al*., 2013) and when completely broken down to glyoxylate releases ammonium ions as by‐products which is then utilized during remobilization or periods of high N demand (Muñoz *et al*., 2001; Watanabe *et al*., 2014; Yang and Han, 2004). Transport of the ureides have been well described in legumes which are facilitated by ureide permease (UPS) proteins (Collier and Tegeder, 2012; Desimone *et al*., 2002; Lescano *et al*., 2016; Pelissier *et al*., 2004). Five *UPS* genes were reported in Arabidopsis (Desimone *et al*., 2002; Schmidt *et al*., 2004, 2006), one in French bean (Pelissier *et al*., 2004) and two in soybean (Collier and Tegeder, 2012).

Our study is the first to evaluate the effects of *UPS* overexpression on the allantoin content in cereal crops such as rice. We evaluated an activation tagging line where the rice *ureide permease 1* gene (*OsUPS1*) was highly activated due to a T‐DNA containing a tetramerized 35S enhance sequence inserted at 1.1 kb upstream of the transcriptional start site of *OsUPS1* resulting in allantoin accumulation. In addition, the overexpression of *OsUPS1* gene resulted in the changes in the metabolic profile of panicles as such asparagine and glutamine during the grain filling stage and exhibited improved growth under suboptimal N conditions. Allantoin partitioning was also altered in overexpression plants driven by a *GOS2* promoter (*OsUPS1* ^*GOS2*^) as well as in silencing plants (*OsUPS1* ^*RNAi*^). We demonstrate that *OsUPS1* is beneficial to rice under N‐limited conditions.

## Results

### Characterization of OsUPS1 expression

**Figure 1:**
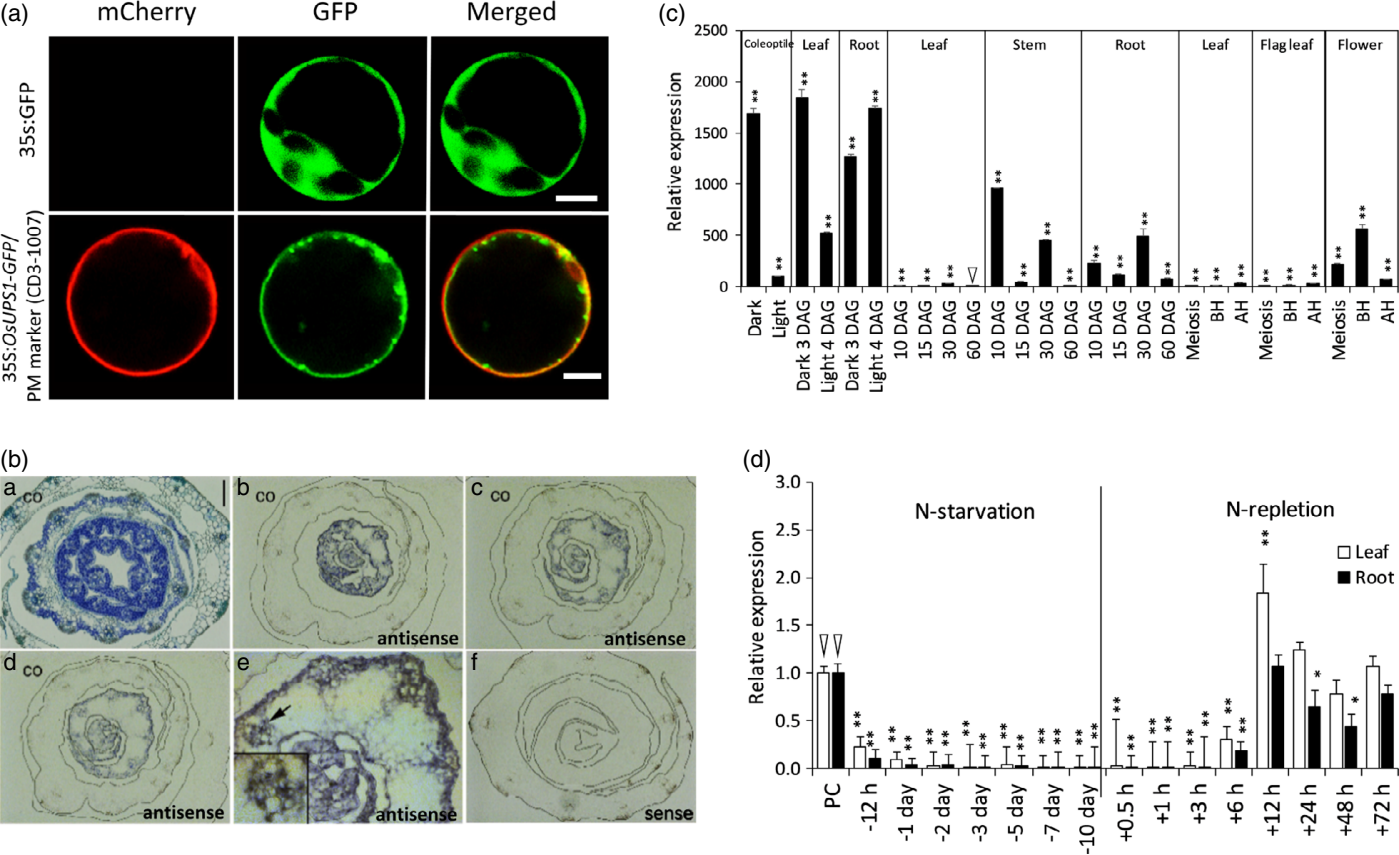
Characterization of rice *OsUPS1* expression. (a) Subcellular localization of OsUPS1 in plasma membranes. Protoplasts were transiently co‐transformed with *35S:OsUPS1‐* GFP and a plasma membrane (PM) marker containing an mCherry fluorescent protein (CD3‐1007, Nelson *et al*., 2007). Fluorescence was observed under a confocal microscope. White bar = 5 μm. (b) *In situ* hybridization on the cross‐section of rice seedlings using *OsUPS1* antisense and sense probes. BA‐ toluidine blue staining; BB‐BD‐ antisense; BE magnified portion, as indicated by a black arrow, of the vasculature having intense signal; BF sense; co‐ Cortex. Black bar = 100 μm. (c) Expression of *OsUPS1* across the developmental stages of rice. Triangle indicates the value used for normalization which showed the lowest *OsUPS1* expression. (d) *OsUPS1* expression in response to changes in exogenous N. Three‐week‐old plants grown in Yoshida solution containing 2.8 mm N ((NH_4_)_2_ SO_4_) were exposed to N‐starvation for 10 days (N‐starvation) and resupplied with the same N‐concentration (N‐repletion). Leaf and root tissues were collected for qRT‐PCR analysis. Values are the means ± SD of three biological samples (*n *=* *3–5) and three technical repeats. Triangles indicate values used for normalization. Asterisks denote significant differences between *OsUPS1* ^*OX*^ and WT by the student's *t*‐test (“*”, *P *<* *0.05; “**”, *P *<* *0.01).

The metabolite allantoin is a potential N source in non‐leguminous plants which can be utilized under N‐limiting conditions, thus we characterized one of its transporters in rice. The rice *ureide permease1* (*OsUPS1*) is a homologue of those found in legumes such as soybean (*GmUPS1*) and French bean (*PvUPS1*) which are known transporters of ureides. To confirm the subcellular localization of OsUPS1, we linked the coding sequence of *OsUPS1* without the stop codon to GFP. The cassette was driven by a 35S promoter and inserted into the pHBT vector (GenBank accession number EF090408) producing the plasmid *35S:OsUPS1‐GFP*. Plasmids were then transiently co‐expressed with a plasma membrane marker (CD3‐1007) (Nelson *et al*., 2007) bearing an mCherry protein in rice protoplasts. Another cassette without the *OsUPS1* gene was used as control producing the plasmid *35S:GFP*. Similar to other reported *UPS* genes, the GFP signal overlapped with the PM marker confirming the transmembrane localization of OsUPS1 (Figure 1a). In addition, *in situ* hybridization analysis on the stem section of 6‐day‐old rice seedlings revealed intense signal around the vascular tissues (Figure 1b) similar to what was observed in soybean (Collier and Tegeder, 2012). Phylogenetic analysis on UPS proteins showed that legumes form a separate clade with non‐leguminous plants such as Arabidopsis and rice (Figure S1). Similar to other UPS proteins, OsUPS1 has the same configuration of transmembrane localization wherein 10 helical domains span across the membrane with a large central loop in the middle (Figure S2).

To characterize the endogenous expression of *OsUPS1*, we extracted total RNA from different tissues collected from different growth stages of rice. qRT‐PCR showed that *OsUPS1* was highly expressed in seedlings younger than 7 days and the expression significantly declined in leaves as the plants mature. The expression of *OsUPS1* was significantly high in roots, stem and flowers compared to leaves (Figure 1c). To determine whether *OsUPS1* is responsive to exogenous N status, 3‐week‐old rice plants (*Oryza sativa* var. Japonica) grown in Yoshida solution containing 2.8 mm N ((NH_4_)_2_SO_4_) were exposed to N‐starvation for 10 days by feeding with Yoshida solution minus (NH_4_)_2_SO_4_. Plants were then resupplied with the same N‐concentration starting on the 11^th^ day. Results showed that transcript levels of *OsUPS1* were quickly down‐regulated within 24 h of N‐starvation and almost no transcripts were detected starting on the 7th day (Figure 1d). When N was re‐introduced, *OsUPS1* expression increased within 12 h suggesting that *OsUPS1* was closely associated with exogenous ammonium status.

### T‐DNA insertion activates expression of OsUPS1

**Figure 2:**
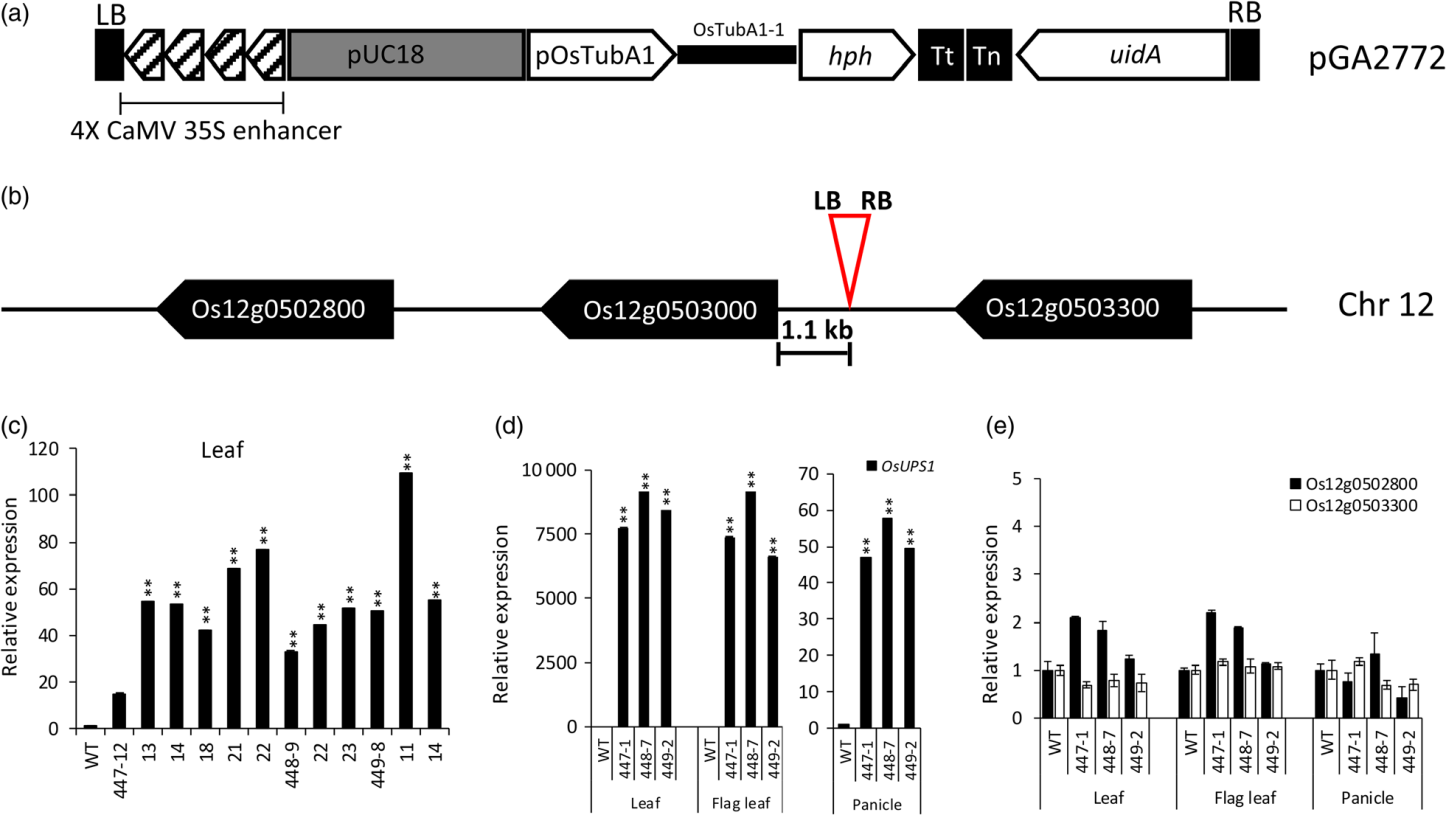
Evaluation of the T‐DNA activation lines. (a) T‐DNA cassette showing the genes present in the activation tagging vector pGA2772 (An *et al*., 2003). The vector contains a tetramerized CaMV 35S enhancer sequence located on the left border responsible for the activation of the adjacent gene. (b) Schematic of the T‐DNA insertion site relative to *OsUPS1* and two other putative *UPS* genes‐ Os12 g0502800 (downstream) and Os12 g0503300 (upstream) in chromosome 12 (Chr 12) of rice. (c) Expression of *OsUPS1* in leaves of 1‐week‐old T_3_ homozygous sister plants. (d–e) Expression of *OsUPS1*, Os12 g0502800 and Os12 g0503300 in leaf tissues and panicles of mature (14 days after flowering (DAF)) activation tagging (*OsUPS1* ^*OX*^) plants. Values are the mean ± SD of three independent experiments. All values were relative to those of WT. Asterisks denote significant differences between *OsUPS1* ^*OX*^ and WT by the student's *t*‐test (**, *P *<* *0.01).

To understand the role of *OsUPS1* in rice, we acquired a T‐DNA activation tagging line kindly provided by Prof. Gynheung An of Kyung Hee University, Korea. The T‐DNA contains a tetramerized 35S enhancer sequence at the left border of the pGA2772 vector (Figure 2a) (An *et al*., 2003). *In silico* BLAT analysis of the flanking sequence showed that the T‐DNA was inserted upstream of *OsUPS1* and the left border was oriented towards the *OsUPS1* gene. To confirm the insertion site, genomic PCR using primers specific to the T‐DNA right border and the plant genome confirmed the insertion of T‐DNA 1.1 kb upstream of the transcriptional start site considered as the promoter region of *OsUPS1* producing the activation tagging plants *OsUPS1* ^*OX*^. In addition, we found two putative *UPS* genes located upstream (Os12 g0503300) and downstream (Os12 g0502800) of *OsUPS1* based on annotation and protein sequence similarity (Figure 2b).

To determine the expression of *OsUPS1* in the activation plants, total RNA from leaves of randomly selected 1‐week‐old homozygous T_3_ *OsUPS1* ^*OX*^ plants was extracted and for qRT‐PCR. Transcript levels of *OsUPS1* were highly up‐regulated in leaves of young plants (Figure 2c) confirming that the 35S enhancer sequence present in the T‐DNA cassette was effective in activating the expression of endogenous *OsUPS1* in *OsUPS1* ^*OX*^ plants. At the reproductive stage 14 days after flowering (14 DAF), we sacrificed three sister lines of *OsUPS1* ^*OX*^ plants as well as WT and collected the flag leaves, leaves (the leaves right below the flag leaf) and panicles. From these tissues, we extracted total RNA qRT‐PCR analysis. Relative to WT, *OsUPS1* expression in leaf, flag leaf and panicle were all highly induced (Figure 2d). To determine whether the enhancer sequence affects the expression of nearby genes, qRT‐PCR was performed on genes Os12 g0502800 (downstream) and Os12 g0503300 (upstream) (Figure 2e). Transcript levels of both genes were not activated indicating that the effect of the enhancer was limited to *OsUPS1* and is a good material for further analysis, thus we further analysed these tissues for metabolite analysis.

### Metabolites are altered in sink tissues of mature OsUPS1^OX^ plants

**Figure 3:**
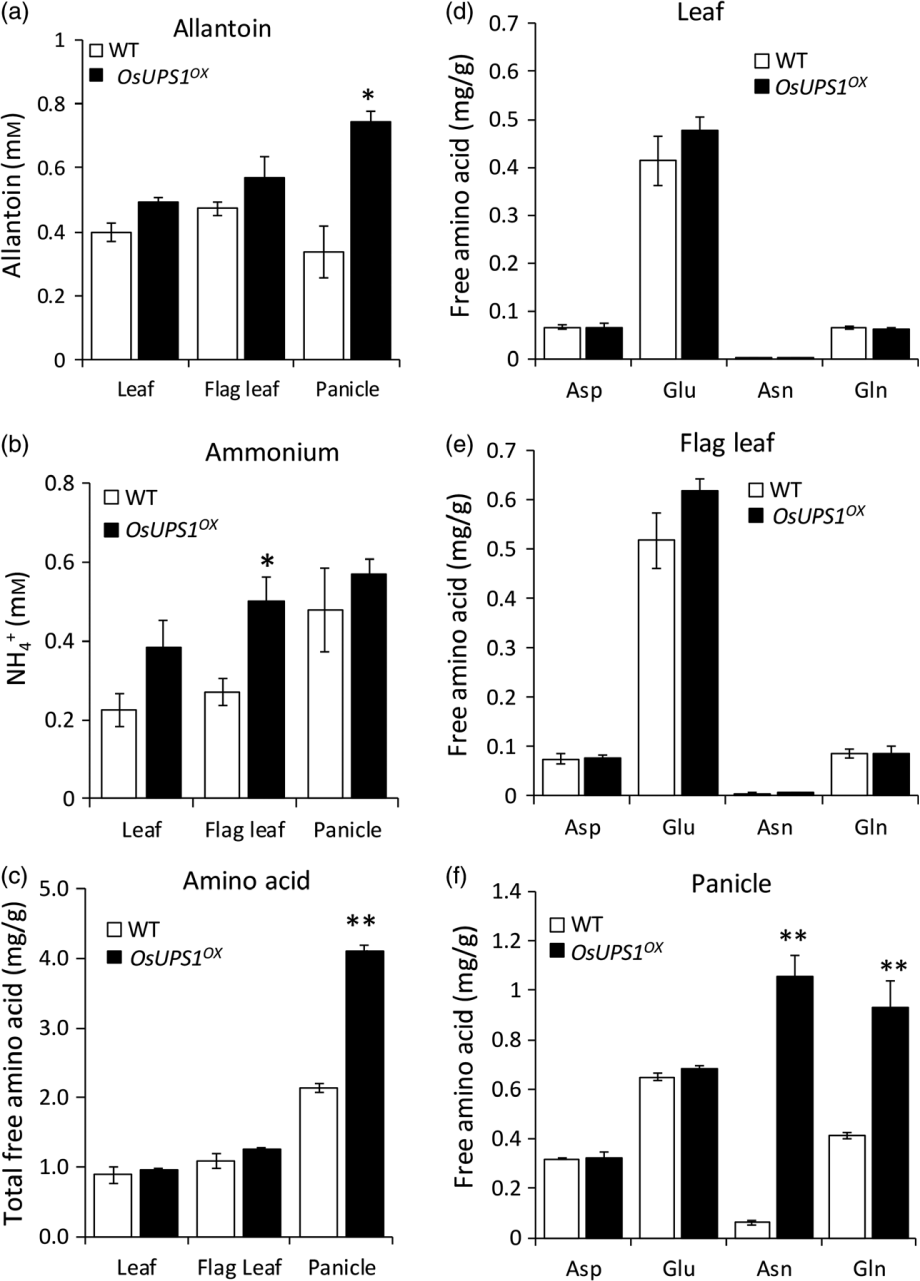
Metabolite analysis on leaf and panicle tissues of 14 DAF plants. Three sister lines were sacrificed and pooled all the leaves right below the flag leaves (leaf), the flag leaves (flag leaf) as well as the panicles from plants grown in a paddy field. Metabolites (a) allantoin, (b) ammonium and (c) total free amino acid were measured in all the tissues. (d–f) Comparison of four amino acids closely linked to N‐assimilation (d) leaf, (e) flag leaf, (f) panicle tissues, respectively. Aspartic acid (Asp); glutamic acid (Glu); asparagine (Asn) and glutamine (Gln). Values are the means ± SD from three biological samples and three technical repeats. Asterisk(s) denotes significant differences between *OsUPS1* ^*OX*^ and WT by the student's *t*‐test (“*”, *P *<* *0.05; “**”, *P *<* *0.01).

To determine the effects of *OsUPS1* overexpression on the endogenous allantoin content in *OsUPS1* ^*OX*^, allantoin was measured in leaf, flag leaf and panicle tissues of 14 DAF plants. Following the overexpression of *OsUPS1*, the endogenous concentration of allantoin in leaves was higher than WT and more significantly in panicles (Figure 3a). Since the sampling time was done during active stage of nutrient remobilization, and since allantoin can serve as ammonium source when catabolized we further measured the ammonium and free amino acids. Ammonium levels in all tissues were relatively higher in *OsUPS1* ^*OX*^ than WT but was significantly higher in flag leaf (Figure 3b). The total free amino acid content in leaf tissues were relatively similar in both plants while in panicles, *OsUPS1* ^*OX*^ showed almost twice to those of WT (Figure 3c, Table S1). The amino acids aspartic acid (Asp), asparagine (Asn), glutamine (Gln) and glutamic acid (Glu), which are closely linked to N‐assimilation, showed relatively similar concentration in leaves of *OsUPS1* ^*OX*^ and WT (Figure 3d–e). In panicles however, we found that the higher total free amino acid content in *OsUPS1* ^*OX*^ was due to the significant accumulation of asparagine and glutamine fractions (Figure 3f). Since glutamine is required for the production of allantoin following ammonium assimilation, we further tested the *OsUPS1* ^*OX*^ plants response to ammonium starvation and repletion at the root level.

### Ammonium level is elevated in roots of OsUPS1 overexpressing plants

**Figure 4:**
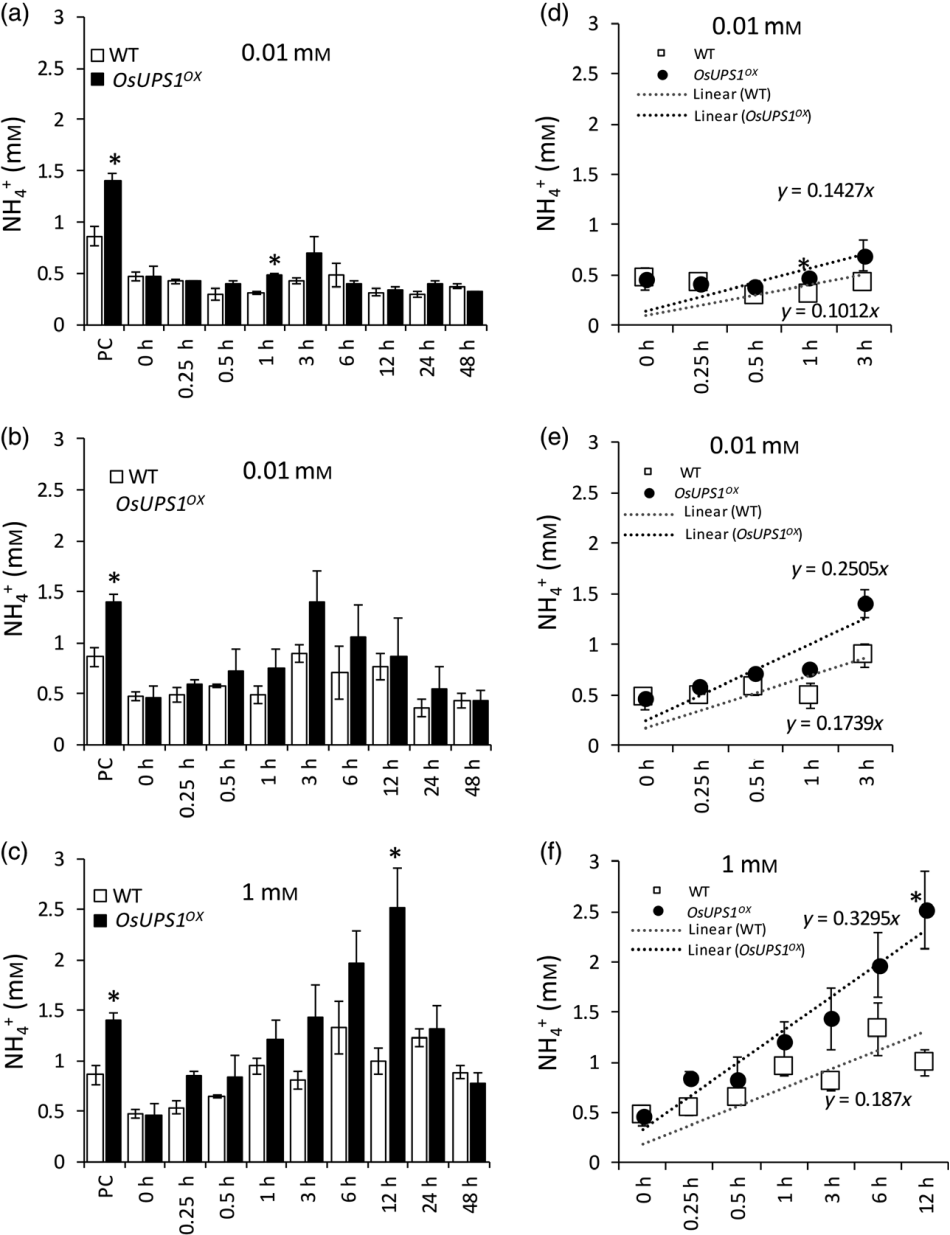
Ammonium content in roots of *OsUPS1* ^*OX*^ and WT at the vegetative stage. Plants were initially grown in Yoshida solution containing 1 mm N ((NH_4_)_2_ SO_4_) for 21 days and subjected to 10 days of N‐starvation by omitting (NH_4_)_2_ SO_4_ in the solution. Three N‐concentrations (0.01, 0.1 and 1 mm N) were then supplied and measured the ammonium content in roots. Concentration of ammonium after resupplying with (a) 0.01, (b) 0.1 and (c) 1 mm concentrations of N, respectively. Slope of ammonium accumulation in roots from the start of feeding up to the peak of accumulation when fed (d) 0.01, (e) 0.1 and (f) 1 mm N, respectively. PC: positive controls. Values are the means ± SD of three biological samples (*n = *5–8) and three technical repeats. Asterisk(s) denote significant differences between *OsUPS1* ^*OX*^ and WT under the same sampling time using the student's *t*‐test (“*”, *P *<* *0.05).

In legumes, allantoin synthesis requires ammonium released during N‐fixation while in non‐leguminous plants, ammonium is imported in roots through the action of ammonium transporters. Since we observed elevated levels of allantoin in sink tissues of mature *OsUPS1* ^*OX*^ plants, we measured the ammonium in roots of plants that were normally fed N, or starved for 10 days and those that were resupplied following starvation. At normal N‐feeding concentration of 1 mm N, ammonium content in *OsUPS1* ^*OX*^ was twice to those of WT while both plants showed relative ammonium level after 10 days of N‐starvation. During N‐repletion, three N‐concentrations were used, that is, 0.01, 0.1 and 1 mm N to determine whether *OsUPS1* ^*OX*^ will show an advantage over WT in terms of ammonium uptake. At ammonium concentration of 0.01 mm, both *OsUPS1* ^*OX*^ and WT plants were relatively similar in ammonium content though at 1 and 3 h after N‐feeding *OsUPS1* ^*OX*^ showed slightly elevated content than WT (Figure 4a). At 0.1 mm N, *OsUPS1* ^*OX*^ showed higher ammonium content with a more pronounced accumulation peak at 3 h compared to WT (Figure 4b). When fed 1 mm N, *OsUPS1* ^*OX*^ showed higher ammonium content than WT reaching a peak at 12 h before depleting back to a concentration close to the control plants suggesting that *OsUPS1* expression affects the ammonium uptake of rice (Figure 4c). These changes in ammonium concentration also showed a more positive slope in *OsUPS1* ^*OX*^ compared to WT under different N‐concentrations (Figure 4 d–f).

### Purine synthesis pathway is induced in OsUPS1^OX^ plants

Since glutamine is used as a substrate for purines synthesis, we measured the transcript levels of enzymes involved in the purine *de novo* synthesis pathway (Stasolla *et al*., 2003) of *OsUPS1* ^*OX*^ roots grown under normal conditions. We found that amido *phosphoribosyltransferases 1, 2* and *3* (*Atase1, 2, 3*), *GAR synthetase* (*GARS*), *GAR formyl transferase* (*GART*), *FGAM synthetase* (*FGAMS*), *AIR synthetase* (*AIRS*), *AIR carboxylase* (*AIRC*), *adenylosuccinate lyase* (*ASL*), *AICAR formyl transferase* (*ATIC*), *SAMP synthetase* (*ASS*), *IMP dehydrogenase* (*IMPDH*) and *GMP synthetase* (*GMPS*) were all highly up‐regulated in *OsUPS1* ^*OX*^ plants compared to WT (Figure S3). These suggest that the purine synthesis pathway was induced in *OsUPS1* ^*OX*^ plants. In addition, the transcript levels of the enzymes *xanthine dehydrogenase* (*XDH*) and *uricase* (*uricase*), which are involved in the conversion of purines to allantoin, did not differ to those of WT (Figure S4) suggesting that the turnover of purines to allantoin was not feedback inhibited, thus allowing allantoin to accumulate. We also found that the C_T_ values of these genes were similar to those of the internal control *OsUbi1* suggesting that the endogenous expression of *XDH* and *uricase* was already very high. It appears then that in rice, the production of allantoin is not solely dependent on the expression of the two key upstream genes for allantoin synthesis but also the expression of the genes in the purine synthesis pathway such that the process leans towards the biosynthesis of allantoin when induced. Furthermore, we measured the transcripts of the enzymes in the allantoin degradation pathway starting from the *Allantoinase* (*ALN*), the key enzyme responsible for the start of allantoin degradation, *Allantoate amidohydrolase* (*AAH*), *Ureidoglycine aminohydrolase* (*UGAH*) and *Ureidoglycolate amidohydrolase* (*UAH*) and found that the transcript levels were relatively similar to WT (Figure S5) indicating that the increase in purine and allantoin synthesis was not coupled with allantoin degradation, thereby allowing it to accumulate.

### Vegetative growth of OsUPS1^OX^ plants is improved under suboptimal N supply

**Figure 5:**
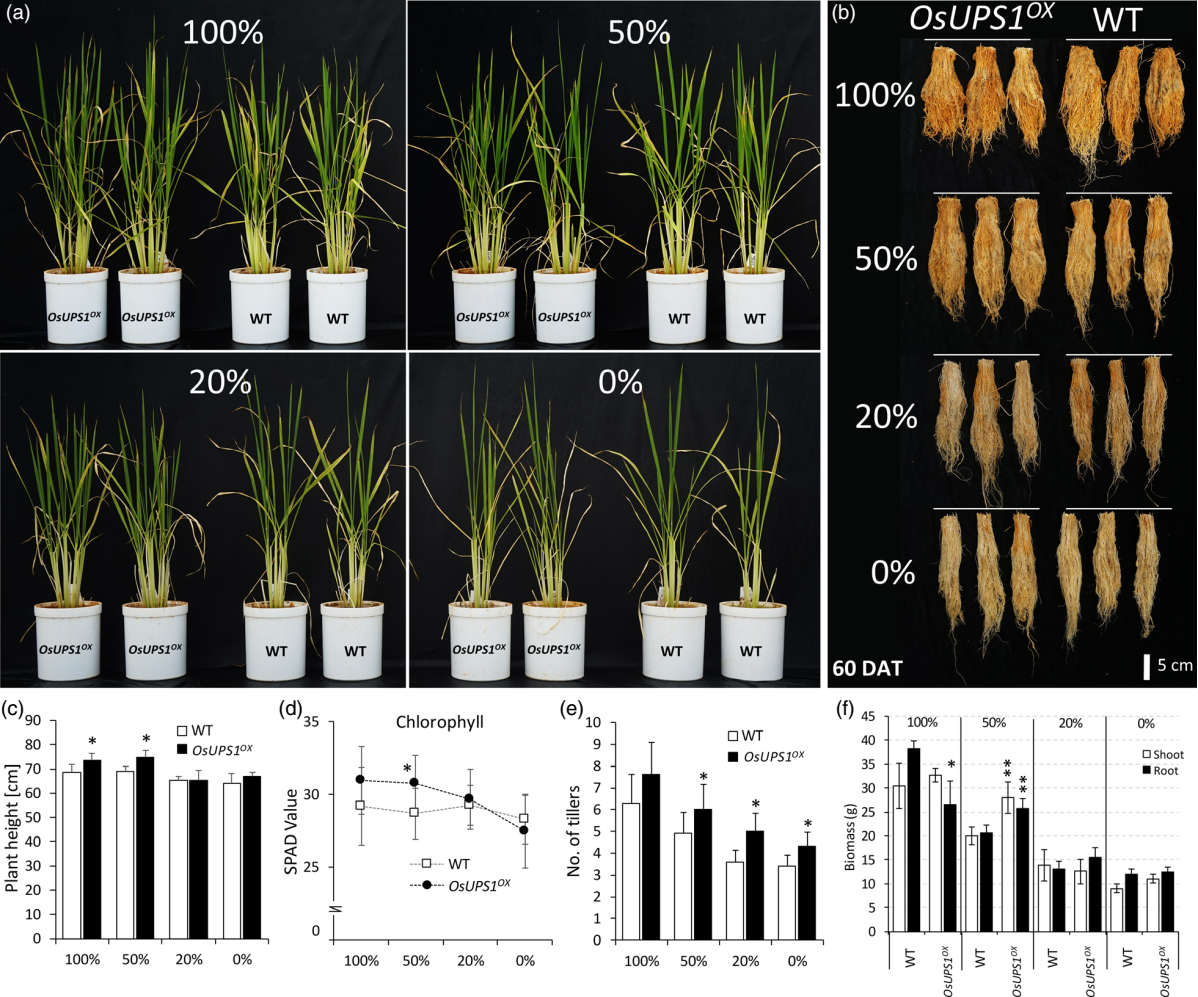
Evaluation of activation tagging plants grown under suboptimal N supply. Uniformly grown 7‐day‐old seedlings were transplanted in a 4‐L pot containing 3.5 kg of low‐N soil. Plants were supplemented with different amount of urea as N source. Morphology of (a) shoots and (b) roots of *OsUPS1* ^*OX*^ in comparison with WT under varying N regime. Measurement of growth traits (c) plant height (d) chlorophyll content represented by SPAD values (e) number of tillers and (f) biomass. Analysis was done on 60 DAT plants. Values are the means ± SD of three biological samples. Asterisk denotes significant differences between *OsUPS1* ^*OX*^ and WT by the student's *t*‐test (“*”, *P *<* *0.05; “**”, *P *<* *0.01).

To determine whether the increased ammonium uptake and elevated allantoin concentration of *OsUPS1* ^*OX*^ plants support plant growth under suboptimal N conditions, we compared its growth with WT under low‐N soil at the vegetative stage. Uniform seedlings of 7‐day‐old *OsUPS1* ^*OX*^ and WT plants pre‐germinated in MS media were transplanted in a 4 L pot containing equal weight of low‐N soil. Plants were then allowed to grow inside a glass house. All pots were rotated twice a week to avoid positional effects. In general, shoot and root phenotype of plants receiving higher N showed better vigour compared to those receiving less (Figure 5 a–b). *OsUPS1* ^*OX*^ was significantly taller than WT under both 100 and 50% N regime (Figure 5c). Chlorophyll content, represented by the SPAD values, was significantly higher in *OsUPS1* ^*OX*^ compared to WT (Figure 5d). In addition, it has been reported that tiller number of rice can give an indication of N availability such that soils with sufficient N result in higher plant tiller number compared to those receiving less (Wada *et al*., 1986). Here, we found that below the 100% N regime, *OsUPS1* ^*OX*^ consistently showed higher number of tillers compared to WT (Figure 5e). In addition, the shoot and root biomass of *OsUPS1* ^*OX*^ at 50% N was 26% and 20% higher than WT, respectively (Figure 5f). Collectively, these suggest that *OsUPS1* ^*OX*^ plants were well adapted in soils having suboptimal N content compared to WT.

### Allantoin partitioning is reflected in constitutive whole‐body overexpression and down‐regulation of OsUPS1

**Figure 6:**
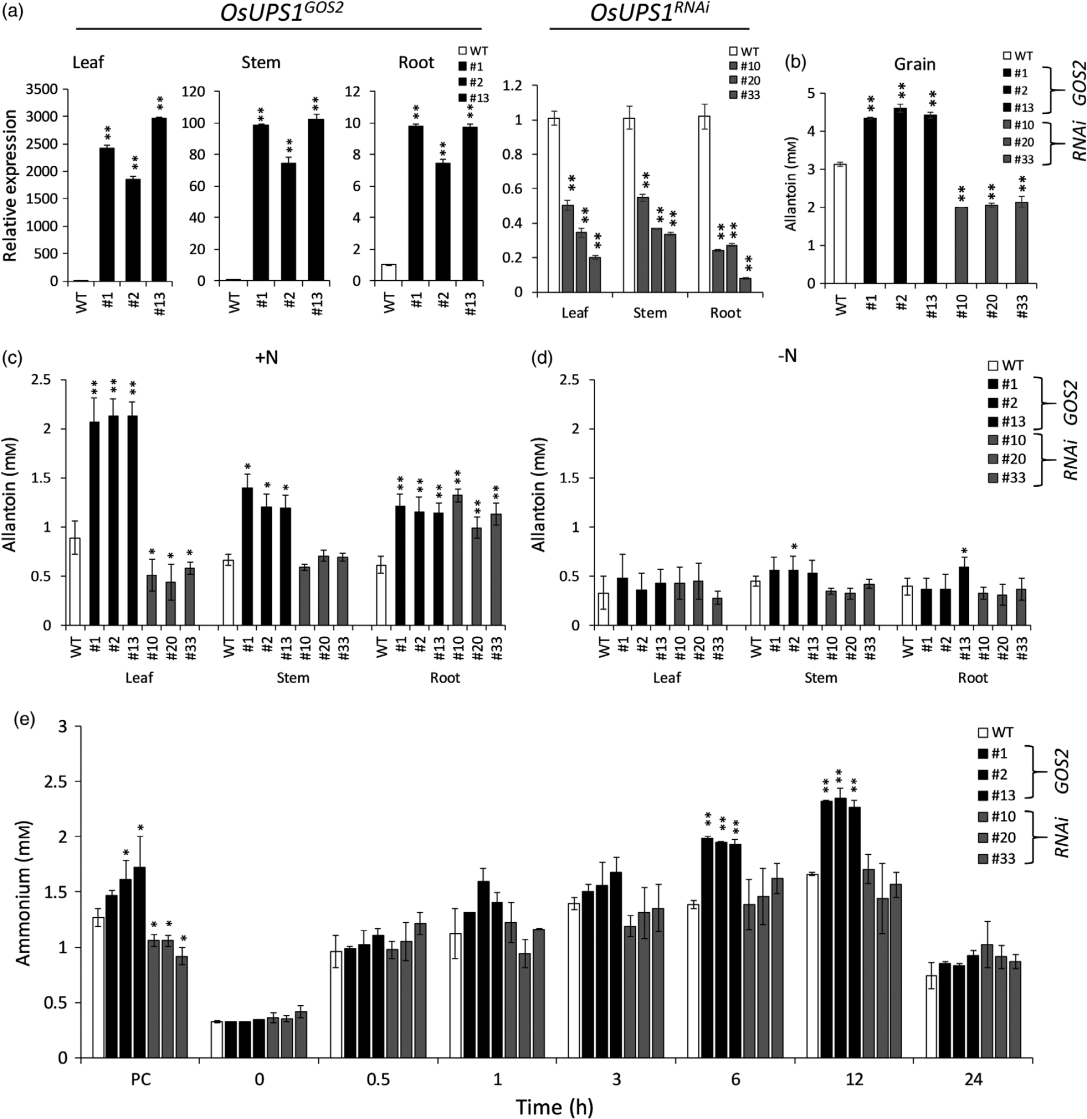
Transcript profile and metabolite quantification of *OsUPS1* ^*GOS2*^ and *OsUPS1* ^*RNAi*^ plants. (a) Relative expression of *OsUPS1* in leaf, stem and root tissues from three independent overexpression *OsUPS1* ^*GOS2*^ (1, 2 and 13) and silencing *OsUPS1* ^*RNAi*^ lines (10, 20 and 33). (b) Allantoin content in grains of T_3_ *OsUPS1* ^*GOS2*^ and *OsUPS1* ^*RNAi*^ as well as WT. (c) Allantoin content in leaves, stems and roots of T_3_ 21‐day‐old *OsUPS1* ^*GOS2*^, *OsUPS1* ^*RNAi*^ as well as WT plants under N‐fed conditions. (d) Allantoin content in plants after 10 days of N‐starvation. (e) Quantification of ammonium in roots of plants under well‐fed (PC), 10‐day starved (0 h) and resupplied with 1 mm N sampled at the indicated time (h). White bars‐ wild type; black bars‐ *OsUPS1* ^*GOS2*^; grey bars‐ *OsUPS1* ^*RNAi*^. Values are the means ± SD of three independent biological samples and three experimental repeats. Asterisk(s) represent significant differences between transgenic plants (*OsUPS1* ^*GOS2*^ and *OsUPS1* ^*RNAi*^) and WT by the student's *t*‐test (“*”, *P *<* *0.05; “**”, *P *<* *0.01).

To further confirm whether the regulation of *OsUPS1* expression alters the allantoin partitioning in rice, we generated another overexpression lines using the whole‐body overexpression *GOS2* promoter (*OsUPS1* ^*GOS2*^ 1, 2, 13) as well as silencing lines for down‐regulation (*OsUPS1* ^*RNAi*^ 10, 20, 33). Transcript levels of *OsUPS1* in three independent homozygous plants showed significant up‐ and down‐regulation of *OsUPS1* in *OsUPS1* ^*GOS2*^ and *OsUPS1* ^*RNAi*^ plants, respectively (Figure 6a). Quantification of allantoin in grains of *OsUPS1* ^*GOS2*^ plants showed significant accumulation similar to what was observed in panicles of *OsUPS1* ^*OX*^ activation lines (Figure 3a) while silencing lines showed significantly lower allantoin content compared to WT (Figure 6b). In addition, tissues from vegetative leaves, stem and roots of *OsUPS1* ^*GOS2*^ lines showed significant increase in allantoin content (Figure 6c). Leaves of *OsUPS1* ^*GOS2*^ plants exhibited the highest increase relative to WT while *OsUPS1* ^*RNAi*^ showed the lowest allantoin content. Interestingly, the roots of *OsUPS1* ^*RNAi*^ showed elevated allantoin relative to WT as a result of the down‐regulation of *OsUPS1* confirming that regulation of *OsUPS1* expression does alter the partitioning of allantoin in rice. To confirm whether allantoin concentration is indeed catabolized during periods of N‐starvation, we exposed the plants to N‐starvation stress for 10 days and collected tissue samples from leaf, stem and roots. After 10 days of N‐starvation, allantoin concentration was reduced in most of the tissues in all plants indicating that the complete removal of N source does allow the plants to utilize allantoin (Figure 6d). These results indicate that rice indeed transport and metabolize allantoin under conditions of high N demand such as during remobilization stage as observed in mature 14 DAF plants and during N‐starvation conditions as tested at the vegetative stage.

We further confirmed in *OsUPS1* ^*GOS2*^ and *OsUPS1* ^*RNAi*^ lines the ammonium content in roots when resupplied with N following 10 days of N‐starvation (Figure 6e). Under normal N‐feeding of 1 mm,* OsUPS1* ^*GOS2*^ plants showed elevated ammonium content while *OsUPS1* ^*RNAi*^ showed lower amount relative to WT. When exposed to complete removal of N source for 10 days, all plants showed similar ammonium level. During N‐repletion with 1 mm N, *OsUPS1* ^*GOS2*^ plants showed a faster accumulation of ammonium similar to what was observed in *OsUPS1* ^*OX*^ activation plants (Figure 4) which also exhibited a peak during 12 h of N‐feeding and subsequently reduced on the 24th h. On the other hand, though *OsUPS1* ^*RNAi*^ showed similar pattern, the ammonium content did not significantly differ with those of WT.

Since there was an increase in ammonium uptake in *OsUPS1* ^*GOS2*^, we further measured the amino acid contents in both roots and leaves of plants. We found a higher free amino acid content in the young leaves of *OsUPS1* ^*GOS2*^ compared to those of *OsUPS1* ^*RNAi*^ and WT (Figure S6). Similar to the panicles of *OsUPS1* ^*OX*^, the increase in the free amino acid content was also due to the asparagine and glutamine fractions. Though there was an increase of glutamine in the sink tissues aboveground, the roots however showed no accumulation of glutamine which suggests that the glutamine pool present in roots was maintained possibly to a homeostatic level. This was probably achieved by readily utilizing glutamine as substrate for purine synthesis while simultaneously transporting some fractions to the shoot as observed in *OsUPS1* overexpressing plants but not in WT and *OsUPS1* ^*RNAi*^ plants, thus avoiding accumulation in roots (Figure S7).

To determine whether the yield was affected in *OsUPS1* ^*GOS2*^ and *OsUPS1* ^*RNAi*^ plants, we scored the grain filling rate under both normally fed (100%) and under N‐limited conditions (50%, 20% and 0% N) Figure S8. We found that among the four N‐feeding regimes, it was at the 20% feeding that *OsUPS1* ^*GOS2*^ started to show a higher filling rate than *OsUPS1* ^*RNAi*^ and WT. In addition, one line from *OsUPS1* ^*GOS2*^ exhibited a highly significant increase in filling rate at 20% N. It was however at 0% N‐feeding that two out of the three *OsUPS1* ^*GOS2*^ lines showed a very significant difference between *OsUPS1* ^*RNAi*^ and WT lines. These observations were even better than what we have observed in *OsUSP1* ^*OX*^ plants which showed growth advantage when N was lowered to 50% N. These results suggest that the increase in internal allantoin concentration due to *OsUPS1* overexpression results in plants that are better adapted in N‐limited growth conditions. Collectively, the overexpression of *OsUPS1* either through gene activation or through the use of an overexpression promoter, can alter the allantoin partitioning and concentration in sink tissues, ammonium uptake in roots, as well as the glutamine fractions in sink tissues resulting in enhanced growth under limited N availability.

## Discussion

In this study, we evaluated the activation tagging plants overexpressing the rice *ureide permease1* (*OsUPS1* ^*OX*^) and showing significant allantoin accumulation in panicles of 14 DAF plants compared to WT (Figure 3a). Though present in rice, the role of allantoin as a N source in rice is not established yet but has been reported to be involved in important biological processes such as stress tolerance (Casartelli *et al*., 2018; Degenkolbe *et al*., 2013; Nam *et al*., 2015) and as a growth enhancer (Wang *et al*., 2012). Allantoin is a stable nitrogen‐rich heterocyclic compound that is a product of purine metabolism and a form N storage in ureide‐metabolizing plants. The synthesis of allantoin in legumes requires ammonium, either acquired from soil or from N‐fixation, which is assimilated into amino acids and ultimately incorporated into amide amino acids (asparagine or glutamine) or ureides (allantoin) for export to leaves (Buchanan *et al*., 2000). When catabolized, allantoin can produce four molar equivalents of ammonium (Zrenner *et al*., 2006) which is re‐assimilated to produce amino acids.

Here, we found that *OsUPS1* expression was regulated by the external N status such that *OsUPS1* was down‐regulated both in roots and leaves during complete removal of N source and rapidly increased when N was resupplied (Figure 1e). A possible explanation is that under normal N conditions, OsUPS1 loads ureides into the xylem for transport to the aerial parts through the transpirational stream similar to what was described by Baral *et al*. (2016). This can be supported by the expression levels of *OsUPS1* being high in roots and stems of plants under sufficient N (Figure 1d), indicative of transport from roots to shoots. However, in absence of N *OsUPS1* was down‐regulated possibly to limit the transport of allantoin to shoot allowing its breakdown to provide ammonium ions in roots. We hypothesize that due to overexpression of *OsUPS1* there was a persistent transport of allantoin away from the source roots and towards the sink shoot tissues leading to accumulation in shoots. Thus, overexpression of *OsUPS1* could provide N in plants by increasing the loading and transport of allantoin to sink tissues where it can be utilized later when the available N in soil becomes limited. In peas, allantoin is transported via the xylem to the leaves where it is either stored or metabolized and loaded into the phloem for transport to the growing parts of the plants (Atkins *et al*., 1982). Subcellular localization showed that OsUSP1 is located in plasma membranes and expressed in vascular tissues including the surrounding companion cells (Figure 1a–b). In French beans, PvUPS1 is expressed in phloem for long distance transport throughout the whole plant (Pelissier and Tegeder, 2007) and in soybean, both GmUPS1‐1 and GmUPS1‐2 are localized in plasma membranes and expressed in nodule cortex and vascular endodermis (Collier and Tegeder, 2012). These suggest that uriedes are transported to locations where N is required either to roots or to shoots. The accumulation of allantoin in panicles of 14 DAF plants suggests roles of allantoin during N remobilization for grain filling. We further confirmed this in grains, leaves, stems and roots of *OsUPS1* ^*GOS2*^ overexpression plants indicating higher loading to sink tissues (Figure 6b–c). Similarly, when the common bean *UPS1* transporter was expressed in cortex and endodermis cells of soybean nodules the delivery of N to shoot was significantly increased improving shoot N nutrition and seed development in legumes (Carter and Tegeder, 2016). In contrast, our *OsUPS1* ^*RNAi*^ silencing lines showed lower allantoin content in similar tissues except in roots where allantoin showed accumulation further indicating that OsUPS1 is required for loading of allantoin to vascular tissues. In soybean, the repressed expression of *GmUPS1* through RNA interference showed accumulation of allantoin and allantoic acid in nodules, a decrease of the ureides in roots and xylem sap and resulted in N deficiency symptoms in leaves (Collier and Tegeder, 2012). We however did not find any N‐deficient symptoms in *OsUPS1* ^*RNAi*^ plants even though the allantoin in shoot was reduced.

In rice, the concentration of allantoin is highest in stem during stem elongation period or panicle initiation to booting stages and decreases dramatically during maturity (Wang *et al*., 2010). This coincide with the reported transport pattern of allantoin where during senescence they are remobilized from vegetative tissues to seeds (Aveline *et al*., 1995; Diaz‐Leal *et al*., 2012; Thomas and Schrader, 1981) and utilized during germination where it is transported from cotyledons to shoot axis (Duran and Todd, 2012; Quiles *et al*., 2009).

Since catabolism of allantoin releases ammonium, these are then utilized for assimilation and synthesis of amino acids (Werner and Witte, 2011). We found significantly high fractions of asparagine and glutamine in panicles of *OsUPS1* ^*OX*^ compared to WT. The concentration of these two amino acids provides information on the N status in plants since they represent the primary amino acids derived from ammonium (Frungillo *et al*., 2014). Soluble asparagine accumulates under a plentiful supply of reduced N (Lea *et al*., 2007) however, stress‐induced accumulation of asparagine is also possible (Lea and Miflin, 2010). Since both *OsUPS1* ^*OX*^ and WT controls were grown in the same cultivating season and paddy field, effects of these stress‐induced factors are minimal, if there is any. It is therefore likely that the altered free amino acid profile in the *OsUPS1* ^*OX*^ was a result of a high supply of reduced N due to the higher ammonium uptake of *OsUPS1* overexpressing plants as shown in the plants response to N‐repletion after being starved for 10 days (Figure 4). We also found that the expression of *OsNRT2.3*, a transporter known to be repressed by elevated ammonium content (Feng *et al*., 2011; Yan *et al*., 2011), was down‐regulated in leaves of *OsUPS1* ^*OX*^ (Figure S9), which was consistent with high level of ammonium in *OsUPS1* ^*OX*^ leaves (Figure 3b). The accumulation of glutamine in panicles could also be a result of high ammonium availability in *OsUPS1* ^*OX*^ compared to WT. In addition, it is well established that both ammonium and glutamine are required for the synthesis of allantoin. For instance, in nodulated plants, bacteroids produce ammonium in nodules that are then transported to the cytosol where assimilation to glutamine occurs. Glutamine is then either transported to the shoot or utilized in the purine *de novo* synthesis pathway in plastids (Shelp and Ireland, 1985) or mitochondria (Atkins and Storer, 1997). In other studies, it was reported that changes in ureide concentration in legumes are reflected in the concentration of glutamine and the response between the metabolites is related (Amarante *et al*., 2006). Similar to legumes, both overexpression lines *OsUPS1* ^*OX*^ and *OsUPS1* ^*GOS2*^ showed higher ammonium content in roots of N‐replete plants following a 10‐day N‐starvation. There was also a significant increase in the transcript levels of the enzymes found in the purine *de novo* synthesis pathway suggesting an increased turnover of glutamine to purines. A recent study by Coleto *et al*. (2016) actually showed that in *P. vulgaris,* PvPRAT3 is responsible for the *de novo* synthesis of purines which in turn is utilized for ureide synthesis. This suggests that changes in the purine synthesis pathway can also affect the downstream ureide metabolism pathway. Generally, plants do not just accumulate purines but readily interconvert them to different biomolecules and one of these is allantoin. When allantoin is produced, it is transported to sink tissues such as the leaves where it can be stored or catabolized to supply N. Thus, the cycle of allantoin production in roots and catabolism in shoot is more pronounced in *OsUPS1* ^*OX*^ than in WT. These suggest that the overexpression of *OsUPS1* can alter the N status of plants through enhanced production and translocation of allantoin.

Varying the exogenous N levels also influenced the overall vitality of plants as manifested when *OsUPS1* ^*OX*^ and WT plants were grown in soil with suboptimal N‐concentrations. We observed that *OsUPS1* ^*OX*^ seemed to show an advantage over WT by exhibiting overall vigour, taller plant height, higher tiller number and biomass as well as higher SPAD values in soil having 50% N (Figure 5) indicating that overexpression of a ureide transporter can bring benefits to plants under N‐limited conditions. (Tegeder, 2014) has reported that altering the import of amino acids or ureides into the collection phloem could improve biomass of plants at the vegetative stage. Indeed, we found that overexpression of *OsUPS1* resulted in higher plant biomass at 50% N‐feeding. In addition, the roots of *OsUPS1* ^*OX*^ plants fed 100% N showed reduced length compared to WT which is a general response of plants fed sufficient ammonium supply (Chen *et al*., 2013). Similarly, Hoque *et al*. (2006) reported that transgenic plants overexpressing *OsAMT1‐1* showed increased biomass only during lower ammonium feeding and suggested that the increased ammonium uptake was balanced with ammonium assimilation. The same can be claimed in our results where reducing the N application showed to be more beneficial to *OsUPS1* ^*OX*^ plants than at higher N.

**Figure 7:**
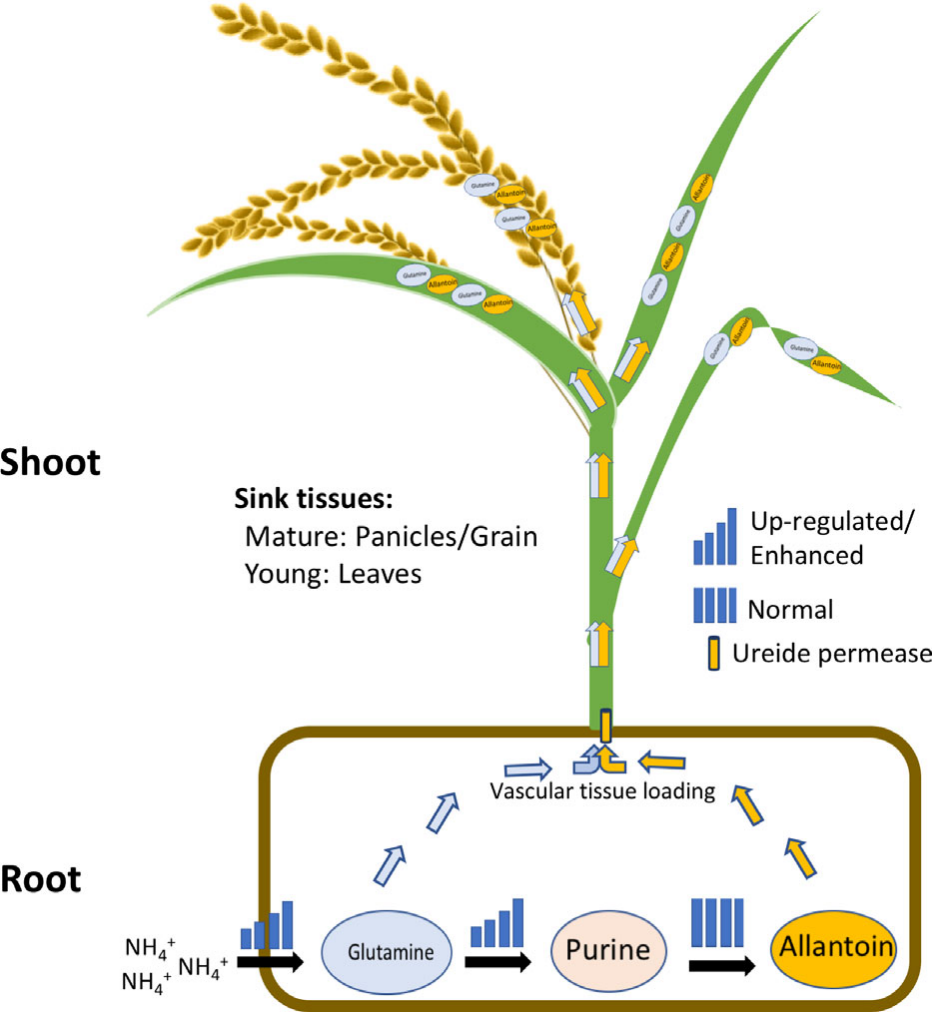
Schematic representation of allantoin partitioning in *OsUPS1*‐overexpressing plants. *OsUPS1*‐overexpressing plants *OsUPS1* ^*OX*^ and *OsUPS1* ^*GOS2*^ both showed higher allantoin content in their sink tissues compared to WT and *OsUPS1* ^*RNAi*^. This can be attributed to the proposed exporter function of Ureide permease1 in roots. The persistent loading of allantoin into the vascular tissues resulted in the increased tendency of plants to synthesize more allantoin in roots. As a result, plants increased its ammonium uptake, thus elevating the glutamine fractions which follows to fates‐ first, transported to shoot where it can accumulate in sink tissues and second, used as substrate for purine and allantoin synthesis.

In summary, we showed that *OsUPS1* overexpression resulted in allantoin accumulation especially in sink tissues such as in grains of mature plants or in leaves and stems of young plants. We also showed that the increase in allantoin production resulted in an elevated ammonium uptake and N‐assimilation to glutamine. Under limited N conditions, plants overexpressing *OsUPS1* showed growth advantage over WT due to the availability of allantoin as an additional N source. Thus, we hypothesize that *OsUPS1* is responsible for exporting allantoin out of the source cells across the plasma membrane and loading into the xylem for transport through the transpirational stream. In addition, the persistent export of allantoin out of the source roots promotes the synthesis of allantoin resulting in elevated allantoin concentration in plants overexpressing *OsUPS1*. We therefore propose a model for the improved growth of *OsUPS1* overexpressing plants under limited N conditions (Figure 7). To produce higher ureide (allantoin) in rice plants that generally adopts the amide strategy for N metabolism, plants must increase the ammonium uptake for assimilation of N (ammonium) to glutamine. Glutamine then proceeds to two fates—first is the immediate transport to sink tissues where it can accumulate either in leaves of young plants or in grains of mature plants or second, serves as the substrate for purine synthesis leading to the synthesis of allantoin. Therefore, the persistent transport of allantoin in roots to shoots promoted the *OsUPS1* overexpressing plants to uptake more ammonium leading to increased glutamine fractions, activation of purine synthesis pathway, and increased production of allantoin which in turn conferred growth advantage to the plants grown under limited N conditions.

## Experimental procedures

### Plant material, genotyping and rice transformation

T‐DNA insertion line (PFG_2D‐4064.L) was provided by Prof. Gynheung An of Kyunghee University and amplified on a rice paddy field. In this study, T_3_ seeds were planted on a rice paddy field at Kyungpook National University, Gunwi (128:34E/36:15N), Korea. Wild type (WT) *Oryza sativa L. ssp. japonica* cv. Dongjin and *OsUPS1* ^*OX*^ seedlings were transplanted into pots 25 days after sowing. Fertilizer was applied at 70N/40P/70K kg/ha after the last paddling and 45 days after transplantation. For genotyping, genomic DNA extraction was performed using DNeasy 96 Plant Kit (QIAGEN, Valencia, CA), according to manufacturer's instructions. T‐DNA insertion was then confirmed through PCR using the primers listed in Table S2.

To generate *GOS2:OsUPS1* overexpression plants (*OsUPS1* ^*GOS2*^), the coding sequence of *OsUPS1* (Os12 g0503000) was amplified from rice cDNA (*Oryza sativa L. ssp. japonica* cv. Nipponbare) using a high‐fidelity DNA polymerase PrimeStar (TaKaRa, Japan). The amplified fragment was subcloned into the rice transformation vector *p700*‐GOS2 (Jeong *et al*., 2010) which carries the 2.2‐kb promoter on the upstream region of *GOS2* (Os07 g0529800) (Pater *et al*., 1992) using the Gateway system (Invitrogen, Carlsbad, CA) (Figure S10). To generate silencing lines named *OsUPS1* ^*RNAi*^, 327 bp from the coding region (between −183 bp and +144 bp from the TGA stop codon) was cloned into two sites separated by a GUS sequence of the *p700‐*GOS2‐RNAi vector (Lee *et al*., 2016) through Gateway system (Invitrogen, Carlsbad, CA). Plasmids were introduced into *Agrobacterium tumefaciens* LBA4404 through triparental mating and embryonic calli from rice seeds (*Oryza sativa L. ssp. japonica* cv. Dongjin) were transformed as previously described (Jang *et al*., 1999). Copy number were determined in T0 plants through Taq‐Man PCR as described by (Bang *et al*., 2015) and single‐copy plants were selected and propagated in a rice paddy field at Kyungpook National University, Gunwi (128:34E/36:15N), Korea. T_3_ plants were used for further analysis.

### Ammonium uptake assay

For ammonium uptake, T_4_ *OsUPS1* ^*OX*^ and WT plants were grown in a 10 L tank containing Yoshida solution (Yoshida *et al*., 1976) for 3 weeks. The solution was changed every 3 days to ensure consistent supply of nutrients. On the 4^th^ week, plant roots were washed with running water and plants were transferred to a new tank containing Yoshida solution without N and allowed to grow for 10 more days. After 10 days of N‐starvation, ammonium sulphate corresponding to three N‐concentrations (0.01, 0.1 and 1 mm N) was introduced. Before sampling, roots were washed with water containing 1 mm CaSO_4_ for 1 min to remove any ammonium present on the surface of roots. For confirmation using different overexpression system, three independent homozygous T_3_ *OsUPS1* ^*GOS2*^ (1, 2, 13) and *OsUPS1* ^*RNAi*^ (10, 20, 33) as well as WT plants were subjected to the same stress as the *OsUPS1* ^*OX*^ plants. Samples were ground in liquid nitrogen and kept in a −80 °C freezer until use.

### Metabolite analysis

Amino acid quantification was sent to National Instrumentation Center for Environmental Management (NICEM) in Seoul National University, Korea. Analysis was done using HPLC Ultimate 3000 equipped with column VD Spher 100 C18‐E (4.6 mm × 150 mm, 3.5 μm/VDS, Optilab, Germany) and FL detector 1260 FLD (Agilent Technologies, Santa Clara, CA, USA) according to manufacturer's manual. For allantoin quantification, samples were analysed based on differential chemical hydrolysis of ureides and measured using a spectrophotometer (NanoQuant, Infinite M200, Switzerland) at 535 nm wavelength as described by (Vogels and Van der Drift, 1970). Concentration was measured according to the standard curves for allantoin (Sigma‐Aldrich, Korea), allantoic acid (TRC, Canada) and glyoxylate standards (Sigma‐Aldrich, Korea).

For ammonium analysis, the Berthelot reaction was followed with some modifications. Pre‐weighed ground samples were first extracted with 2% sulpho‐salisylic acid and passed through a syringe filter (ADVANTEC, Toyo Roshi Kaisha LTD., Japan). The reagents were Reagent A (0.33 m sodium phenolate in 2 N NaOH with pH adjusted to pH 13), Reagent B (0.01% sodium nitroprussiate in water) and Reagent C (26 mm sodium hypochlorite). Ten μL of each extract was reacted with 50 μL of each of the reagents in a sequential manner in triplicates. The reaction was performed in a 96 well plate (SPL Life Sciences, Korea) for 1 h and read through a spectrophotometer (NanoQuant, Infinite M200, Switzerland) at 635 nm wavelength. Results were then compared through a standard curve using ammonium sulphate (Sigma‐Aldrich, http://www.sigmaaldrich.com) as standard compound. For glutamine analysis, the glutamine analysis kit from (USBiological Life Sciences, Marblehead, MA, USA) was performed according the manufacturer's instructions.

### Subcellular localization and microscopy

Leaf sheaths from 50 etiolated rice seedlings were cut into 1‐ to 2‐ mm pieces using a fresh sharp razor blade on a glass plate. The leaf sheath pieces were quickly transferred to 10 mL digestion solution containing 0.6 m mannitol, 0.6 mm 4‐morpholineethanesulfonic acid (MES), 1.5% cellulase RS (Yakult, Japan), 0.75% macerozyme (Yakult, Japan), 1 mm CaC1_2_, 0.1% Bovine Serum Albumin (BSA), 5 mm beta‐mercaptoethanol, pH 5.7. Samples were incubated for 4–5 h in dark with gentle shaking (40–50 rpm) at 28 °C and subjected to vacuum infiltration every hour. After incubation, the digestion solution containing the protoplasts was collected in 50 mL tube. Subsequent washing using 10 mL (twice) of W5 solution (154 mm NaCl, 125 mm CaC1_2_, 5 mm KC1, 5 mm D‐glucose 2 mm MES, pH 5.7) was done and pooled the collected protoplasts. The suspension was passed through 70 μm and 40 μm nylon mesh. The pooled suspension was centrifuged at 320 ***g*** for 8 min and washed three times with W5 solution. The pellet was resuspended in MMg solution (4 mm MES, pH 5.7, 0.6 m mannitol, and 15 mm MgCl_2_). Protoplasts were quantified by microscopy using a haemocytometer before transformation. The *35S:OsUPS1‐GFP* plasmid were co‐transformed with mCherry protein marker specific for plasma membrane (CD3‐1008) (Nelson *et al*., 2007) to protoplasts using PEG‐mediated transformation. After 12 h incubation at 28 °C, the protoplasts were harvested by centrifugation at 300 ***g*** for 2 min and viewed using Leica SP8 STED laser scanning confocal microscope (Leica). Images were processed using Leica LAS AF Lite Software. GFP was excited at 488 nm and the emitted light was detected between 512 and 560 nm. RFP was excited at 555 and emission was detected between 584 and 610 nm.

### Phenotypic and agronomic trait analysis under suboptimal N supply

A total of 40 uniformly grown seedlings for each *OsUPS1* ^*OX*^ and WT plants were transplanted in a 4‐L pot filled with equal weight (3.5 kg) of low‐N soil containing 0.023% (w/w) total nitrogen (TN) which was four times lower than ordinary nursery soil (0.101% (w/w) TN). Soil was excavated from a hill (2 metres deep) in Yongin City, Korea (127:12E/37:7N). Ten plants were used for each N level of 100, 50, 20 and 0% N. The concentration of 100% N is equal to 0.215 g of urea supplied per pot. During the tillering stage, 30% of the initial N was supplied and another 20% during the booting stage. All pots were supplemented once with 0.48 and 0.19 g of phosphorus and potassium, respectively (Farm Hannong Co., Ltd., Korea). Images were taken using Sony Alpha α5000 camera (SONY CORP., Japan). Chlorophyll was measured using SPAD (KONICA MINOLTA INC., Japan). For the filling rate of *OsUPS1* ^*GOS2*^ and *OsUPS1* ^*RNAi*^, similar procedures were done except that the plants were grown in a rain‐off shelter at Kyungpook National University, Gunwi (128:34E/36:15N), Korea.

### Quantitative real‐time PCR analysis

RNeasy plant mini‐kit (QIAGEN) was used to isolate total RNA from all the tissues mentioned in this study. For cDNA synthesis, 2000 ng of total RNA was used as initial template and the RevertAid^TM^ First Strand cDNA Synthesis Kit (Fermentas, Burlington, Canada) was used for cDNA synthesis. Real‐time PCR analysis was performed using the Mx3000P Real‐Time PCR system (Stratagene, La Jolla, CA). Reactions were performed at 98 °C for 10 min, followed by 40 cycles of 98 °C for 20 s, 60 °C for 40 s, 72 °C for 20 s in a 20 μL volume mix containing 1 μL EvaGreen™ Mix (SolGent, Deajeon, Korea), 0.25 μm primers and 20 ng cDNA. Primers were designed using the Primer‐BLAST designing tool (www.ncbi.nlm.nih.gov) and listed in Table S2. To ensure measurement of only the required PCR product at a specific melting point, a melting curve analysis was performed at 55–95 °C at a heating rate of 0.1 °C/s according to the procedure of (Park *et al*. 2010). *OsUbi1* gene (AK121590) was used as an internal standard. Relative gene expression was calculated through the 2^−Δ ΔCT^ method by Livak and Schmittgen (2001). Values are the means ± SD (standard deviation) of three biological samples and three experimental repeats.

### *In Situ* hybridization

*In situ* hybridization experiments were performed as previously described (Lee *et al*., 2003), with minor modifications. Briefly, wild type rice plants *Oryza sativa L. ssp. japonica* cv. Ilmi were grown on MS media for 6 days and stem sections were fixed in a solution containing 50% ethanol, 5% acetic acid and 3.7% formaldehyde. They were then embedded in paraplast (Sigma, St Louis,MO) and 8 μm cross‐sectional cuts were made using a microtome. Digoxigenin (DIG)‐labelled *OsUPS1* antisense and sense probes were generated through *in vitro* transcription from the 5′UTR to the coding region (1.4 kb) with DIG‐labelled UTP (Roche, Mannheim, Germany) using the SP6 RNA polymerase and the T7 RNA polymerase, respectively (Roche, Mannheim, Germany). Paraplast were removed and the sections were then dried on a slide warmer and incubated in a humidified box containing one of the probes (0.8 μg/slide) for 12 h. After hybridization, sections were washed and colour‐reacted in a solution containing blue tetrazolium chloride (NBT), 5‐bromo‐4‐chloro‐3‐indolyl‐phosphate (BCIP) and levamisole for 36 h in a dark humidified box and mounted with Permount^®^ (Fisher Scientific, Fair Lawn, NJ). Photographs were taken under a bright field illumination using a microscope (ZEISS AXIO Imager.A2, Germany) equipped with a camera (ZEISS Axiocam 506 Color, Germany).

## Conflicts of interest

The authors declare no conflicts of interest.

## Supporting information


**Figure S1** Phylogenetic analysis of UPS proteins from leguminous and non‐leguminous plants.
**Figure S2** Topology of UPS from different plant species.
**Figure S3** Transcript levels of enzymes in the purine synthesis pathway.
**Figure S4** Transcript levels of enzymes in the allantoin synthesis pathway.
**Figure S5** Transcript levels of enzymes in the allantoin degradation pathway.
**Figure S6** Free amino acid contents in *OsUPS1* ^*GOS2*^ and *OsUPS1* ^*RNAi*^ plants.
**Figure S7** Glutamine concentration in roots of *OsUPS1* ^*OX*^ plants after resupplying N.
**Figure S8** Filling rate of *OsUPS1* ^*GOS2*^ and *OsUPS1* ^*RNAi*^ plants grown under different N‐concentrations.
**Figure S9** Expression of *OsNRT2.3* in leaf tissues of 14 DAF *OsUPS1* ^*OX*^ plants.
**Figure S10** Vectors used for rice transformation with overexpression and silence cassettes.
**Table S1** Free amino acid content in leaf, flag leaf and panicles of 14 DAF plants.
**Table S2** List of oligomers used in this study.

## References

[pbi13054-bib-0001] Alamillo, J.M. , Diaz‐Leal, J.L. , Sanchez‐Moran, M.V. and Pineda, M. (2010) Molecular analysis of ureide accumulation under drought stress in *Phaseolus vulgaris* L. Plant, Cell Environ. 33, 1828–1837. 20545885 10.1111/j.1365-3040.2010.02187.x

[pbi13054-bib-0002] Amarante, L.D. , Lima, J.D. and Sodek, L. (2006) Growth and stress conditions cause similar changes in xylem amino acids for different legume species. Environ. Exp. Bot. 58, 123–129.

[pbi13054-bib-0100] An, S. , Park, S. , Jeong, D.‐H. , Lee, D.‐Y. , Kang, H.‐G. , Yu, J.‐H. , Hur, J. , *et al*. (2003) Generation and analysis of end sequence database for T‐DNA tagging lines in rice. Plant Physiol. 133, 2040–2047. 14630961 10.1104/pp.103.030478 PMC300755

[pbi13054-bib-0003] Atkins, C.A. and Storer, P. (1997) Reexamination of the intracellular localization of *de novo* purine synthesis in cowpea nodules. Plant Physiol. 113, 127–135. 12223595 10.1104/pp.113.1.127 PMC158123

[pbi13054-bib-0004] Atkins, C.A. , Pate, J.S. , Ritchie, A. and Peoples, M.B. (1982) Metabolism and Translocation of Allantoin in Ureide‐Producing Grain Legumes. Plant Physiol. 70, 476–482. 16662519 10.1104/pp.70.2.476 PMC1067173

[pbi13054-bib-0005] Aveline, A. , Crozat, Y. , Pinochet, X. , Domenach, A.‐M. and Cleyet‐Marel, J.‐C. (1995) Early remobilization: a possible source of error in the ureide assay method for N2 fixation measurement by early maturing soybean. Soil Sci. Plant Nutr. 41, 737–751.

[pbi13054-bib-0006] Bang, S.W. , Park, S.‐H. , Kim, Y.S. , Do Choi, Y. and Kim, J.‐K. (2015) The activities of four constitutively expressed promoters in single‐copy transgenic rice plants for two homozygous generations. Planta, 241, 1529–1541. 25809149 10.1007/s00425-015-2278-4

[pbi13054-bib-0007] Baral, B. , da Silva, J.A.T. and Izaguirre‐Mayoral, M.L. (2016) Early signaling, synthesis, transport and metabolism of ureides. J. Plant Physiol. 193, 97–109. 26967003 10.1016/j.jplph.2016.01.013

[pbi13054-bib-0008] Brychkova, G. , Alikulov, Z. , Fiuhr, R. and Sagi, M. (2008) A critical role for ureides in dark and senescence‐induced purine remobilization is unmasked in the Atxdh1 Arabidopsis mutant. Plant J. 54, 496–509. 18266920 10.1111/j.1365-313X.2008.03440.x

[pbi13054-bib-0009] Buchanan, B.B. , Gruissem, W. and Jones, R.L. (2000) Biochemistry & Molecular Biology of Plants, Rockville, MD: American Society of Plant Physiologists.

[pbi13054-bib-0010] Carter, A.M. and Tegeder, M. (2016) Increasing nitrogen fixation and seed development in soybean requires complex adjustments of nodule nitrogen metabolism and partitioning processes. Curr. Biol. 26, 2044–2051. 27451897 10.1016/j.cub.2016.06.003

[pbi13054-bib-0011] Casartelli, A. , Riewe, D. , Hubberten, H.M. , Altmann, T. , Hoefgen, R. and Heuer, S. (2018) Exploring traditional aus‐type rice for metabolites conferring drought tolerance. Rice, 11, 9. 29372429 10.1186/s12284-017-0189-7 PMC5785456

[pbi13054-bib-0012] Chen, G. , Guo, S. , Kronzucker, H.J. and Shi, W. (2013) Nitrogen use efficiency (NUE) in rice links to NH4 + toxicity and futile NH4 + cycling in roots. Plant Soil, 369, 351–363.

[pbi13054-bib-0013] Coleto, I. , Trenas, A.T. , Erban, A. , Kopka, J. , Pineda, M. and Alamillo, J.M. (2016) Functional specialization of one copy of glutamine phosphoribosyl pyrophosphate amidotransferase in ureide production from symbiotically fixed nitrogen in *Phaseolus vulgaris* . Plant, Cell Environ. 39, 1767–1779. 27004600 10.1111/pce.12743

[pbi13054-bib-0014] Collier, R. and Tegeder, M. (2012) Soybean ureide transporters play a critical role in nodule development, function and nitrogen export. Plant J. 72, 355–367. 22725647 10.1111/j.1365-313X.2012.05086.x

[pbi13054-bib-0015] Degenkolbe, T. , Do, P.T. , Kopka, J. , Zuther, E. , Hincha, D.K. and Köhl, K.I. (2013) Identification of Drought Tolerance Markers in a Diverse Population of Rice Cultivars by Expression and Metabolite Profiling. PLoS ONE, 8, e63637. 23717458 10.1371/journal.pone.0063637 PMC3661581

[pbi13054-bib-0016] Desimone, M. , Catoni, E. , Ludewig, U. , Hilpert, M. , Schneider, A. , Kunze, R. , Tegeder, M. *et al*. (2002) A novel superfamily of transporters for allantoin and other oxo derivatives of nitrogen heterocyclic compounds in Arabidopsis. Plant Cell, 14, 847–856. 11971139 10.1105/tpc.010458 PMC150686

[pbi13054-bib-0017] Diaz‐Leal, J.L. , Galvez‐Valdivieso, G. , Fernandez, J. , Pineda, M. and Alamillo, J.M. (2012) Developmental effects on ureide levels are mediated by tissue‐specific regulation of allantoinase in *Phaseolus vulgaris* L. J. Exp. Bot. 63, 4095–4106. 22442417 10.1093/jxb/ers090

[pbi13054-bib-0018] Duran, V.A. and Todd, C.D. (2012) Four allantoinase genes are expressed in nitrogen‐fixing soybean. Plant Physiol. Biochem. 54, 149–155. 22476036 10.1016/j.plaphy.2012.03.002

[pbi13054-bib-0019] Feng, H. , Yan, M. , Fan, X. , Li, B. , Shen, Q. , Miller, A.J. and Xu, G. (2011) Spatial expression and regulation of rice high‐affinity nitrate transporters by nitrogen and carbon status. J. Exp. Bot. 62, 2319–2332. 21220781 10.1093/jxb/erq403

[pbi13054-bib-0020] Frungillo, L. , Skelly, M.J. , Loake, G.J. , Spoel, S.H. and Salgado, I. (2014) S‐nitrosothiols regulate nitric oxide production and storage in plants through the nitrogen assimilation pathway. Nat. Commun. 5, 5401. 25384398 10.1038/ncomms6401 PMC4229994

[pbi13054-bib-0021] Gruber, N. and Galloway, J.N. (2008) An Earth‐system perspective of the global nitrogen cycle. Nature, 451, 293–296. 18202647 10.1038/nature06592

[pbi13054-bib-0022] Han, M. , Okamoto, M. , Beatty, P.H. , Rothstein, S.J. and Good, A.G. (2015) The Genetics of Nitrogen Use Efficiency in Crop Plants. Annu. Rev. Genet. 49(49), 269–289. 26421509 10.1146/annurev-genet-112414-055037

[pbi13054-bib-0023] Hanks, J.F. , Tolbert, N.E. and Schubert, K.R. (1981) Localization of Enzymes of Ureide Biosynthesis in Peroxisomes and Microsomes of Nodules. Plant Physiol. 68, 65–69. 16661891 10.1104/pp.68.1.65 PMC425890

[pbi13054-bib-0024] Hoque, M.S. , Masle, J. , Udvardi, M.K. , Ryan, P.R. and Upadhyaya, N.M. (2006) Over‐expression of the rice OsAMT1‐1 gene increases ammonium uptake and content, but impairs growth and development of plants under high ammonium nutrition. Funct. Plant Biol. 33, 153–163. 32689222 10.1071/FP05165

[pbi13054-bib-0025] Jang, I.‐C. , Nahm, B.H. and Kim, J.‐K. (1999) Subcellular targeting of green fluorescent protein to plastids in transgenic rice plants provides a high‐level expression system. Mol. Breed. 5, 453–461.

[pbi13054-bib-0026] Jeong, J.S. , Kim, Y.S. , Baek, K.H. , Jung, H. , Ha, S.‐H. , Do Choi, Y. , Kim, M. *et al*. (2010) Root‐specific expression of OsNAC10 improves drought tolerance and grain yield in rice under field drought conditions. Plant Physiol. 153, 185–197. 20335401 10.1104/pp.110.154773 PMC2862432

[pbi13054-bib-0027] Layzell, D.B. and Larue, T.A. (1982) Modeling C and N Transport to Developing Soybean Fruits. Plant Physiol. 70, 1290–1298. 16662669 10.1104/pp.70.5.1290 PMC1065877

[pbi13054-bib-0028] Lea, P.J. and Miflin, B.J. (2010) Nitrogen Assimilation and its Relevance to Crop Improvement. In Annual Plant Reviews, vol. 42, pp. 1–40. Hoboken, NJ: Wiley‐Blackwell.

[pbi13054-bib-0029] Lea, P.J. , Sodek, L. , Parry, M.A.J. , Shewry, R. and Halford, N.G. (2007) Asparagine in plants. Ann. Appl. Biol. 150, 1–26.

[pbi13054-bib-0101] Lee, D.K. , Ahn, J.H. , Song, S.K. , Choi, Y.D. and Lee, J.S. (2003) Expression of an expansin gene is correlated with root elongation in soybean. Plant Physiol. 131, 985–997. 12644651 10.1104/pp.009902 PMC166864

[pbi13054-bib-0030] Lee, D.‐K. , Jung, H. , Jang, G. , Jeong, J.S. , Kim, Y.S. , Ha, S.‐H. , Do Choi, Y. *et al*. (2016) Overexpression of the OsERF71 transcription factor alters rice root structure and drought resistance. Plant Physiol. 172, 575–588. 27382137 10.1104/pp.16.00379 PMC5074616

[pbi13054-bib-0031] Lescano, C.I. , Martini, C. , Gonzalez, C.A. and Desimone, M. (2016) Allantoin accumulation mediated by allantoinase downregulation and transport by Ureide Permease 5 confers salt stress tolerance to Arabidopsis plants. Plant Mol. Biol. 91, 581–595. 27209043 10.1007/s11103-016-0490-7

[pbi13054-bib-0032] Livak, K.J. and Schmittgen, T.D. (2001) Analysis of relative gene expression data using the real‐time quantitative PCR and the 2‐Δ Δ CT method. Methods, 25, 402–408. 11846609 10.1006/meth.2001.1262

[pbi13054-bib-0033] Matsumoto, T. , Yatazawa, M. and Yamamoto, Y. (1977) Distribution and change in the contents of allantoin and allantoic acid1 in developing nodulating and non‐nodulatng soybean plants. Plant Cell Physiol. 18, 353–359.

[pbi13054-bib-0034] Mulvaney, R.L. , Khan, S.A. and Ellsworth, T.R. (2009) Synthetic nitrogen fertilizers deplete soil nitrogen: a global dilemma for sustainable cereal production. J. Environ. Qual. 38, 2295–2314. 19875786 10.2134/jeq2008.0527

[pbi13054-bib-0035] Muñoz, A. , Piedras, P. , Aguilar, M. and Pineda, M. (2001) Urea Is a Product of Ureidoglycolate Degradation in Chickpea. Purification and Characterization of the Ureidoglycolate Urea‐Lyase. Plant Physiol. 125, 828–834. 11161040 10.1104/pp.125.2.828 PMC64884

[pbi13054-bib-0036] Nam, M.H. , Bang, E. , Kwon, T.Y. , Kim, Y. , Kim, E.H. , Cho, K. , Park, W.J. *et al*. (2015) Metabolite Profiling of Diverse Rice Germplasm and Identification of Conserved Metabolic Markers of Rice Roots in Response to Long‐Term Mild Salinity Stress. Int. J. Mol. Sci. 16, 21959–21974. 26378525 10.3390/ijms160921959 PMC4613291

[pbi13054-bib-0037] Nelson, B.K. , Cai, X. and Nebenfuhr, A. (2007) A multicolored set of *in vivo* organelle markers for co‐localization studies in Arabidopsis and other plants. Plant J. 51, 1126–1136. 17666025 10.1111/j.1365-313X.2007.03212.x

[pbi13054-bib-0038] Park, S.‐H. , Yi, N. , Kim, Y.S. , Jeong, M.H. , Bang, S.W. , Choi, Y.D. and Kim, J.K. (2010) Analysis of five novel putative constitutive gene promoters in transgenic rice plants. J. Exp. Bot. 61, 2459–2467. 20363869 10.1093/jxb/erq076 PMC2877896

[pbi13054-bib-0039] Pate, J.S. , Atkins, C.A. , White, S.T. , Rainbird, R.M. and Woo, K.C. (1980) Nitrogen Nutrition and Xylem Transport of Nitrogen in Ureide‐Producing Grain Legumes. Plant Physiol. 65, 961–965. 16661314 10.1104/pp.65.5.961 PMC440456

[pbi13054-bib-0040] Pater, B.S. , Mark, F. , Rueb, S. , Katagiri, F. , Chua, N.H. , Schilperoort, R.A. and Hensgens, L.A. (1992) The promoter of the rice gene GOS2 is active in various different monocot tissues and binds rice nuclear factor ASF‐1. Plant J. 2, 837–844. 1302635 10.1111/j.1365-313x.1992.00837.x

[pbi13054-bib-0041] Pelissier, H.C. and Tegeder, M. (2007) PvUPS1 plays a role in source‐sink transport of allantoin in French bean (*Phaseolus vulgaris*). Funct. Plant Biol. 34, 282–291. 32689354 10.1071/FP06277

[pbi13054-bib-0042] Pelissier, H.C. , Frerich, A. , Desimone, M. , Schumacher, K. and Tegeder, M. (2004) PvUPS1, an allantoin transporter in nodulated roots of French bean. Plant Physiol. 134, 664–675. 14764906 10.1104/pp.103.033365 PMC344542

[pbi13054-bib-0043] Quiles, F.A. , Raso, M.J. , Pineda, M. and Piedras, P. (2009) Ureide metabolism during seedling development in French bean (*Phaseolus vulgaris*). Physiol. Plant. 135, 19–28. 19121096 10.1111/j.1399-3054.2008.01173.x

[pbi13054-bib-0044] Rainbird, R.M. , Thorne, J.H. and Hardy, R.W.F. (1984) Role of Amides, Amino‐Acids, and Ureides in the Nutrition of Developing Soybean Seeds. Plant Physiol. 74, 329–334. 16663418 10.1104/pp.74.2.329 PMC1066678

[pbi13054-bib-0045] Sagi, M. , Omarov, R.T. and Lips, S.H. (1998) The Mo‐hydroxylases xanthine dehydrogenase and aldehyde oxidase in ryegrass as affected by nitrogen and salinity. Plant Sci. 135, 125–135.

[pbi13054-bib-0046] Schmidt, A. , Su, Y.H. , Kunze, R. , Warner, S. , Hewitt, M. , Slocum, R.D. , Ludewig, U. *et al*. (2004) UPS1 and UPS2 from Arabidopsis mediate high affinity transport of uracil and 5‐fluorouracil. J. Biol. Chem. 279, 44817–44824. 15308648 10.1074/jbc.M405433200

[pbi13054-bib-0047] Schmidt, A. , Baumann, N. , Schwarzkopf, A. , Frommer, W.B. and Desimone, M. (2006) Comparative studies on Ureide Permeases in Arabidopsis thaliana and analysis of two alternative splice variants of AtUPS5. Planta, 224, 1329–1340. 16738859 10.1007/s00425-006-0315-z

[pbi13054-bib-0048] Shelp, B.J. and Ireland, R.J. (1985) Ureide metabolism in leaves of nitrogen‐fixing soybean plants. Plant Physiol. 77, 779–783. 16664133 10.1104/pp.77.3.779 PMC1064600

[pbi13054-bib-0049] Stasolla, C. , Katahira, R. , Thorpe, T.A. and Ashihara, H. (2003) Purine and pyrimidine nucleotide metabolism in higher plants. J. Plant Physiol. 160, 1271–1295. 14658380 10.1078/0176-1617-01169

[pbi13054-bib-0050] Streeter, J.G. (1979) Allantoin and Allantoic Acid in Tissues and Stem Exudate from Field‐grown Soybean Plants. Plant Physiol. 63, 478–480. 16660751 10.1104/pp.63.3.478 PMC542854

[pbi13054-bib-0051] Tegeder, M. (2014) Transporters involved in source to sink partitioning of amino acids and ureides: opportunities for crop improvement. J. Exp. Bot. 65, 1865–1878. 24489071 10.1093/jxb/eru012

[pbi13054-bib-0052] Thomas, R.J. and Schrader, L.E. (1981) The Assimilation of Ureides in Shoot Tissues of Soybeans. 1. Changes in Allantoinase Activity and Ureide Contents of Leaves and Fruits. Plant Physiol. 67, 973–976. 16661804 10.1104/pp.67.5.973 PMC425812

[pbi13054-bib-0053] Todd, C.D. , Tipton, P.A. , Blevins, D.G. , Piedras, P. , Pineda, M. and Polacco, J.C. (2006) Update on ureide degradation in legumes. J. Exp. Bot. 57, 5–12. 16317038 10.1093/jxb/erj013

[pbi13054-bib-0054] Vogels, G.D. and Van der Drift, C. (1970) Differential analyses of glyoxylate derivatives. Anal. Biochem. 33, 143–157. 5413235 10.1016/0003-2697(70)90448-3

[pbi13054-bib-0055] Wada, G. , Shoji, S. and Mae, T. (1986) Relation between nitrogen absorption and growth and yield of rice plants. Jpn. Agric. Res. Q. 20, 127–145.

[pbi13054-bib-0056] Wang, P. , Kong, C.H. , Sun, B. and Xu, X.H. (2010) Allantoin‐induced changes of microbial diversity and community in rice soil. Plant Soil, 332, 357–368.

[pbi13054-bib-0057] Wang, P. , Kong, C.H. , Sun, B. and Xu, X.H. (2012) Distribution and Function of Allantoin (5‐Ureidohydantoin) in Rice Grains. J. Agric. Food Chem. 60, 2793–2798. 22369364 10.1021/jf2051043

[pbi13054-bib-0058] Watanabe, S. , Matsumoto, M. , Hakomori, Y. , Takagi, H. , Shimada, H. and Sakamoto, A. (2014) The purine metabolite allantoin enhances abiotic stress tolerance through synergistic activation of abscisic acid metabolism. Plant, Cell Environ. 37, 1022–1036. 24182190 10.1111/pce.12218

[pbi13054-bib-0059] Werner, A.K. and Witte, C.P. (2011) The biochemistry of nitrogen mobilization: purine ring catabolism. Trends Plant Sci. 16, 381–387. 21482173 10.1016/j.tplants.2011.03.012

[pbi13054-bib-0060] Werner, A.K. , Romeis, T. and Witte, C.‐P. (2010) Ureide catabolism in Arabidopsis thaliana and Escherichia coli. Nat. Chem. Biol. 6, 19–21. 19935661 10.1038/nchembio.265

[pbi13054-bib-0061] Werner, A.K. , Medina‐Escobar, N. , Zulawski, M. , Sparkes, I.A. , Cao, F.Q. and Witte, C.P. (2013) The Ureide‐Degrading Reactions of Purine Ring Catabolism Employ Three Amidohydrolases and One Aminohydrolase in Arabidopsis, Soybean, and Rice. Plant Physiol. 163, 672–681. 23940254 10.1104/pp.113.224261 PMC3793049

[pbi13054-bib-0062] Xu, G. , Fan, X. and Miller, A.J. (2012) Plant nitrogen assimilation and use efficiency. Annu. Rev. Plant Biol. 63, 153–182. 22224450 10.1146/annurev-arplant-042811-105532

[pbi13054-bib-0063] Yan, M. , Fan, X. , Feng, H. , Miller, A.J. , Shen, Q. and Xu, G. (2011) Rice OsNAR2.1 interacts with OsNRT2.1, OsNRT2.2 and OsNRT2.3a nitrate transporters to provide uptake over high and low concentration ranges. Plant, Cell Environ. 34, 1360–1372. 21486304 10.1111/j.1365-3040.2011.02335.x

[pbi13054-bib-0064] Yang, J. and Han, K.H. (2004) Functional Characterization of Allantoinase Genes from Arabidopsis and a Nonureide‐Type Legume Black Locust. Plant Physiol. 134, 1039–1049. 14976234 10.1104/pp.103.034637 PMC389928

[pbi13054-bib-0065] Yoshida, S. , Forno, D.A. , Cook, J.H. and Gomez, K.A. (1976) Laboratory Manual for Physiological Studies of Rice. Los Banos, Laguna, Philippines: The International Rice Research Institute.

[pbi13054-bib-0066] Zrenner, R. , Stitt, M. , Sonnewald, U. and Boldt, R. (2006) Pyrimidine and purine biosynthesis and degradation in plants. Annu. Rev. Plant Biol. 57, 805–836. 16669783 10.1146/annurev.arplant.57.032905.105421

