# Supplementary material for: Allantoin accumulation through overexpression of ureide permease1 improves rice growth under limited nitrogen conditions
Source: Plant Biotechnol J. 2019 Feb 4;17(7):1289–301. doi: 10.1111/pbi.13054 (PMC6577366; doi:10.1111/pbi.13054)
Supplement: Supplementary file 1 — Figure S1 Phylogenetic analysis of UPS proteins from leguminous and non‐leguminous plants. Figure S2 Topology of UPS from different plant species. Figure S3 Transcript levels of enzymes in the purine synthesis pathway. Figure S4 Transcript levels of enzymes in the allantoin synthesis pathway. Figure S5 Transcript levels of enzymes in the allantoin degradation pathway. Figure S6 Free amino acid contents in OsUPS1 GOS2 and OsUPS1 RNAi plants. Figure S7 Glutamine concentration in roots of OsUPS1 OX plants after resupplying N. Figure S8 Filling rate of OsUPS1 GOS2 and OsUPS1 RNAi plants grown under different N‐concentrations. Figure S9 Expression of OsNRT2.3 in leaf tissues of 14 DAF OsUPS1 OX plants. Figure S10 Vectors used for rice transformation with overexpression and silence cassettes. Table S1 Free amino acid content in leaf, flag leaf and panicles of 14 DAF plants. Table S2 List of oligomers used in this study. [file PBI-17-1289-s001.pdf]

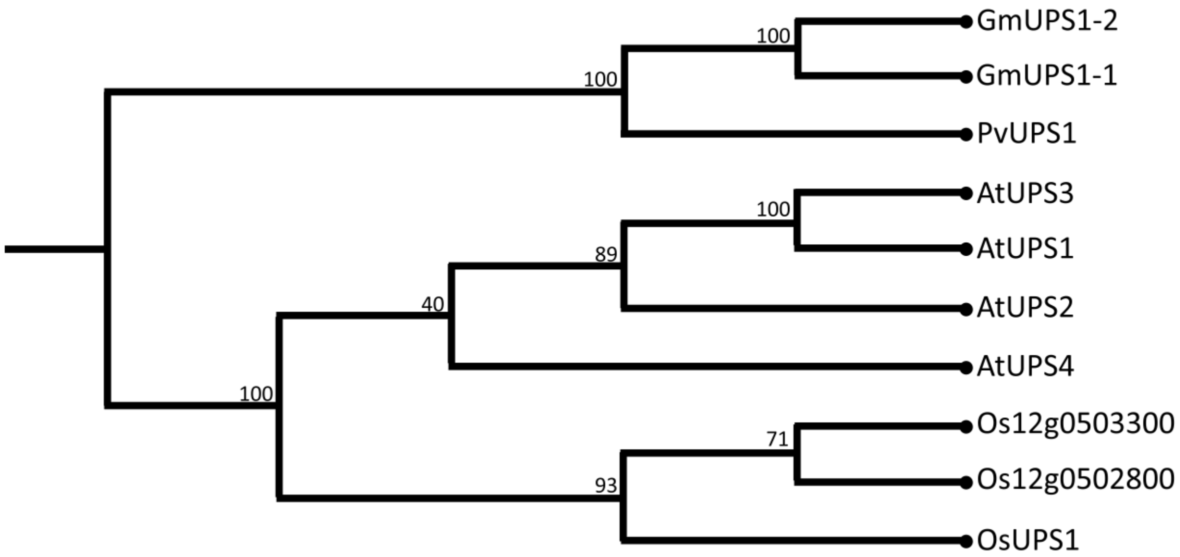

Figure S1. Phylogenetic analysis of UPS proteins from leguminous and non-leguminous plants.

Leguminous plants include soybean (GmUPS1) and French bean (PvUPS1) while non-leguminous plants include Arabidopsis (AtUPS1, AtUPS2, AtUPS3, AtUPS4) and rice OsUPS1 and genes flanking OsUPS1 which is also annotated as UPS proteins in rice- Os12g0502800 (downstream) and Os12g0503300 (upstream). The tree was generated using the CLC Sequence Viewer program. Bootstrap values are indicated in each node

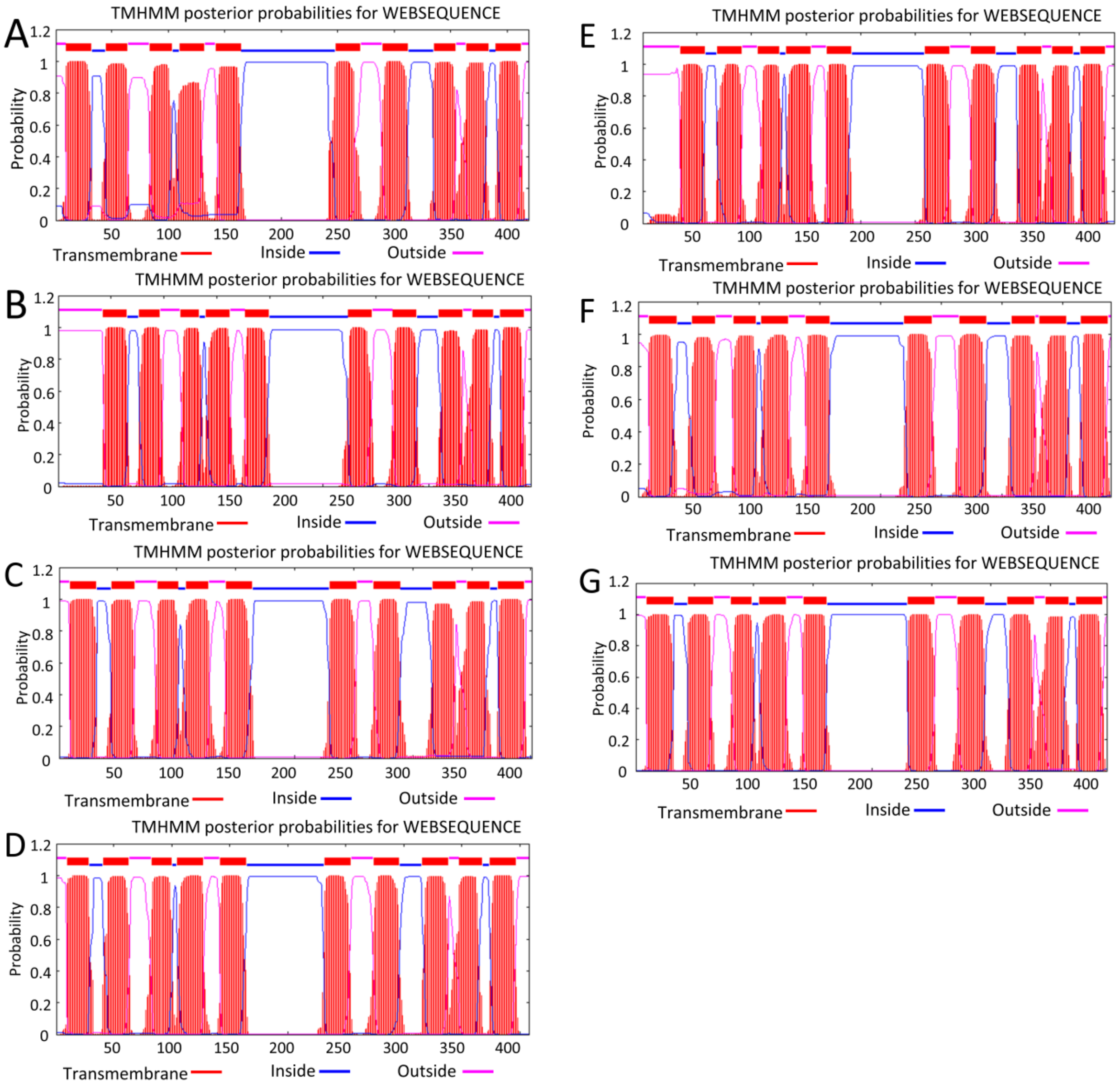

Figure S2. Topology of UPS from different plant species.

(A) OsUPS1 (B) Os12g0502800 (C) Os12g0503300 (D) PvUPS1 (E) GmUPS1-1 (F) AtUPS1 (G) AtUPS2. Simulation was done using the TMHMM web program (http://www.cbs.dtu.dk/services/TMHMM/). Prediction of topology is represented by colors: Red- transmembrane, Blue- inside and Pink- outside.

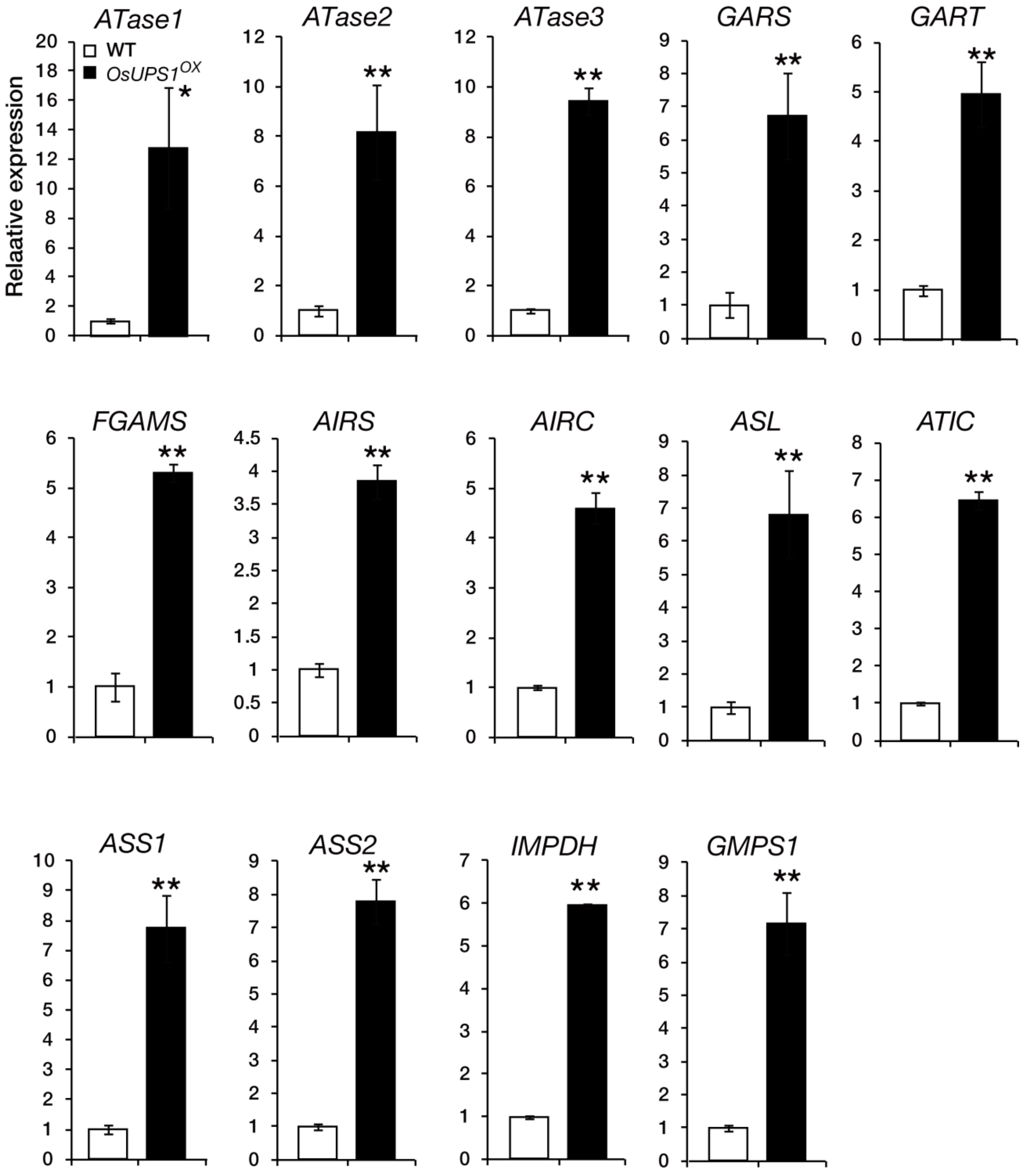

Figure S3. Transcript levels of enzymes in the purine synthesis pathway.

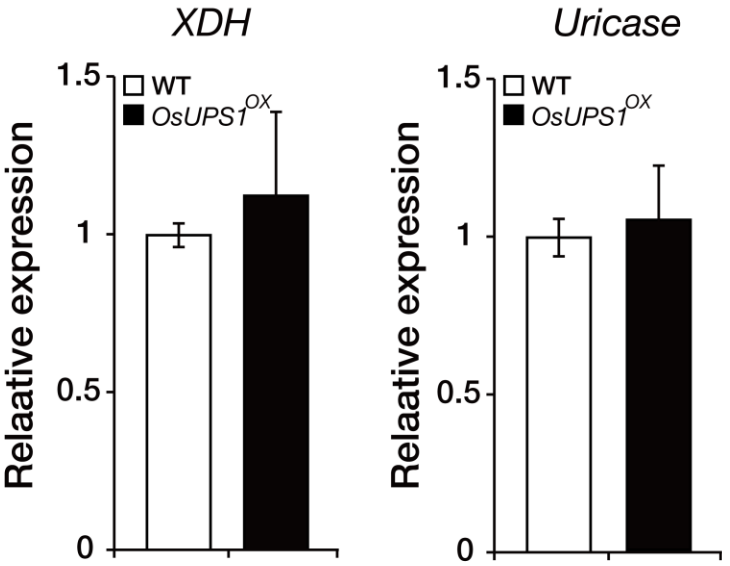

Figure S4. Transcript levels of enzymes in the allantoin synthesis pathway.

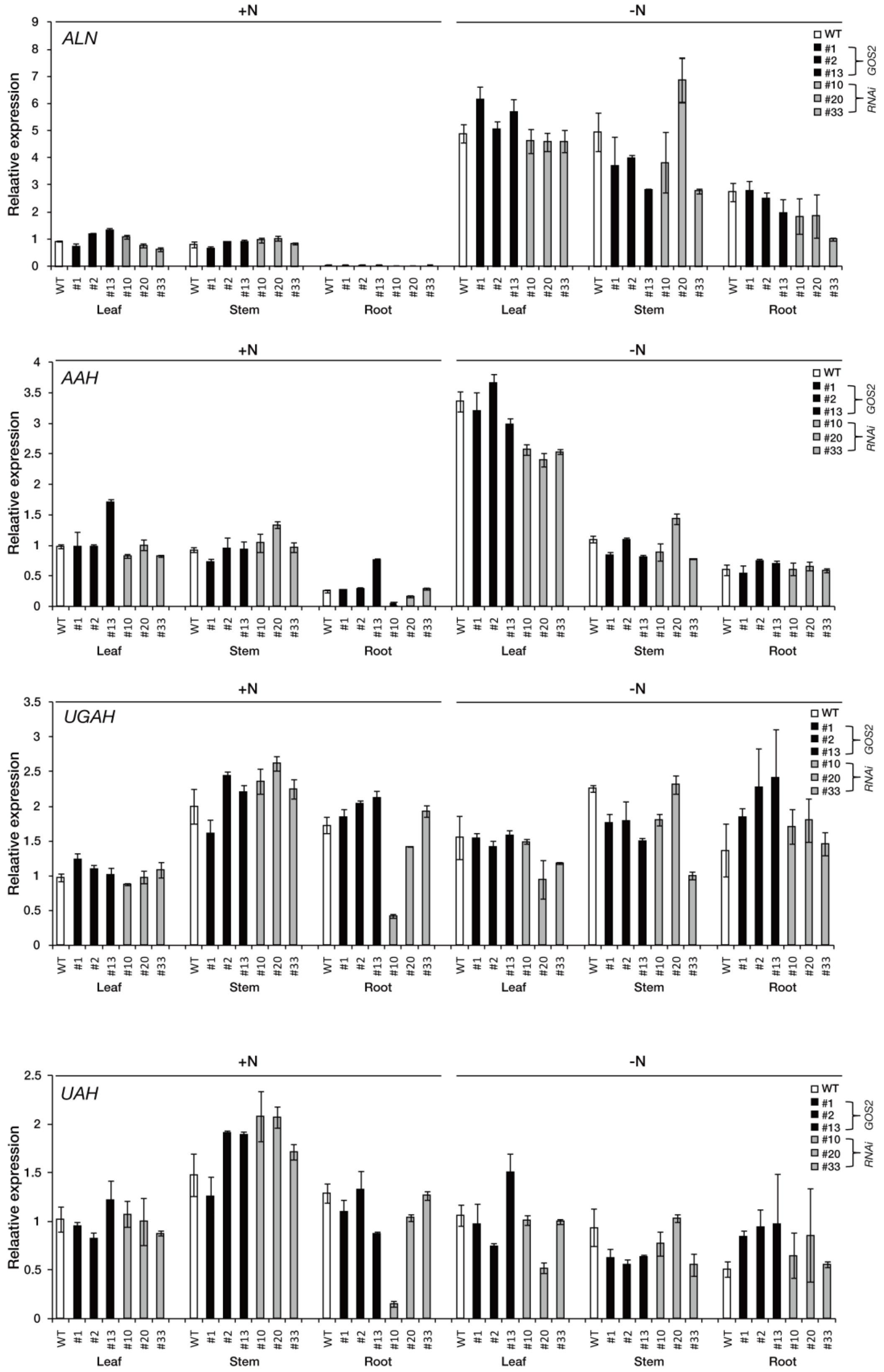

Figure S5. Transcript levels of enzymes in the allantoin degradation pathway.

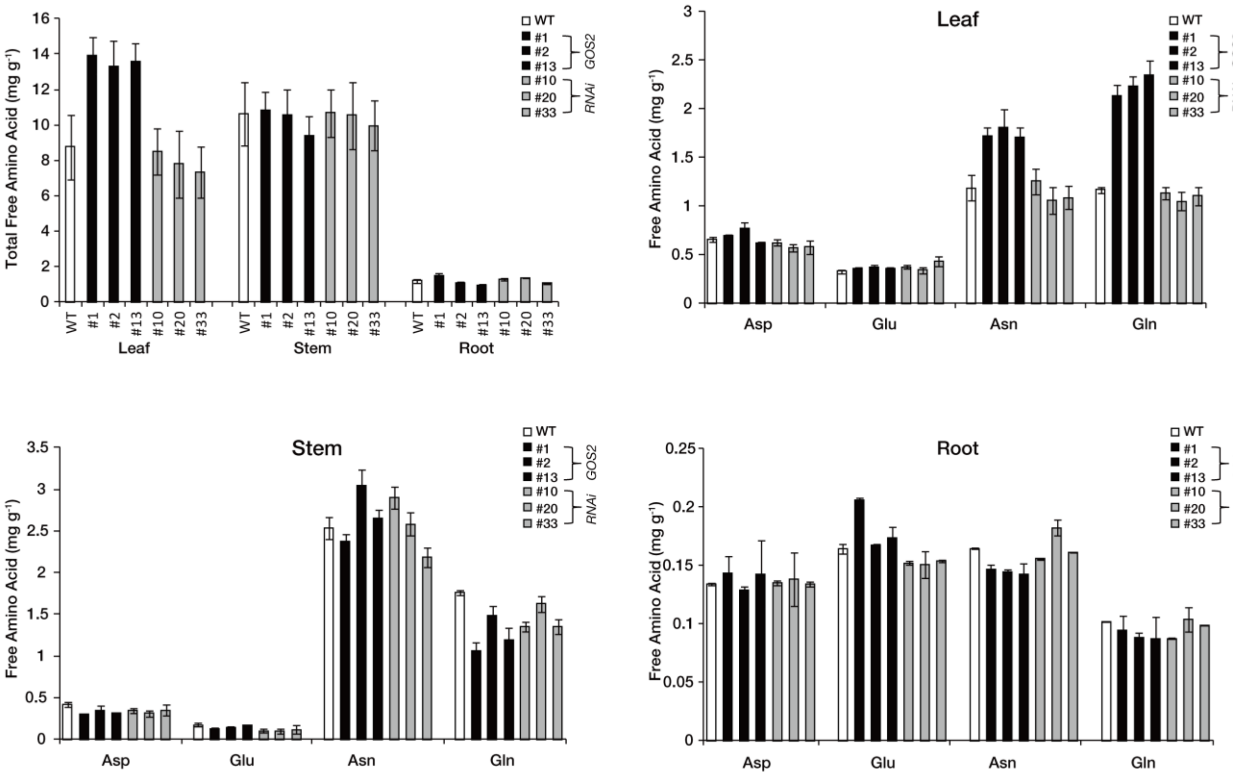

Figure S6. Free amino acid contents in *OsUPS1*^{GOS2} and *OsUPS1*^{RNAi} plants.

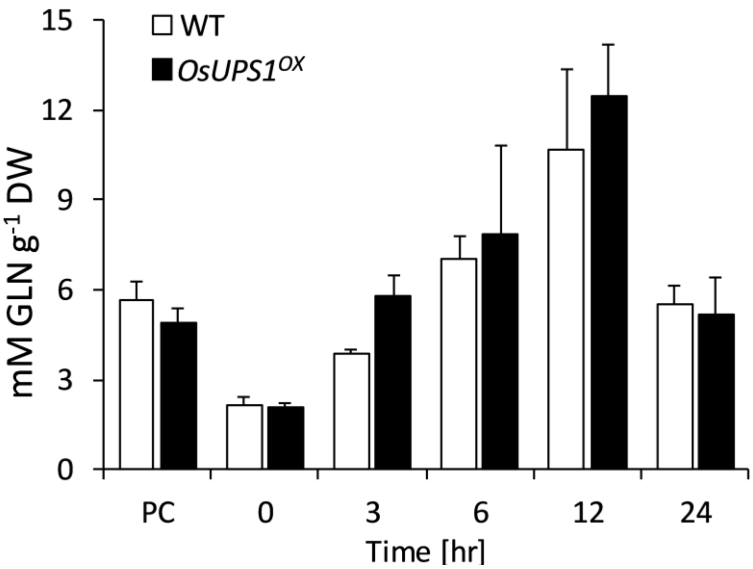

Figure S7. Glutamine concentration in *OsUPS1*^{OX} roots after resupplying N.

Plants initially grown in Yoshida solution for 21 days were subjected to 10 days of N-starvation by excluding $(NH_4)_2SO_4$ in the solution. N concentration of 1 mM N were then resupplied and measured the glutamine content with those of the WT. Values are means ± SD from three biological samples and three technical repeats.

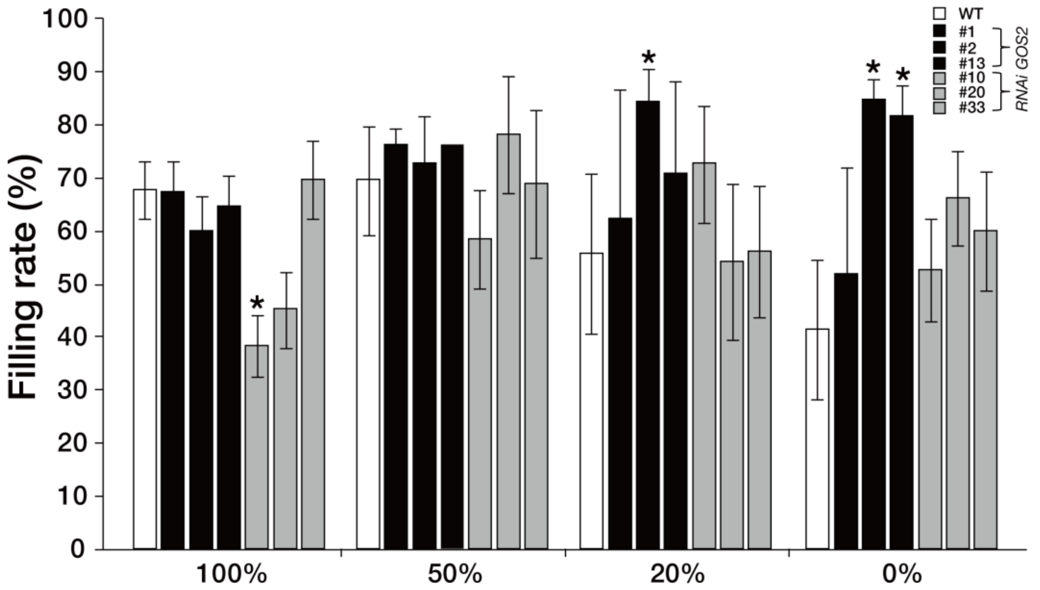

Figure S8. Filling rate of *OsUPS1*^{GOS2} and *OsUPS1*^{RNAi} plants grown under different N-concentrations.

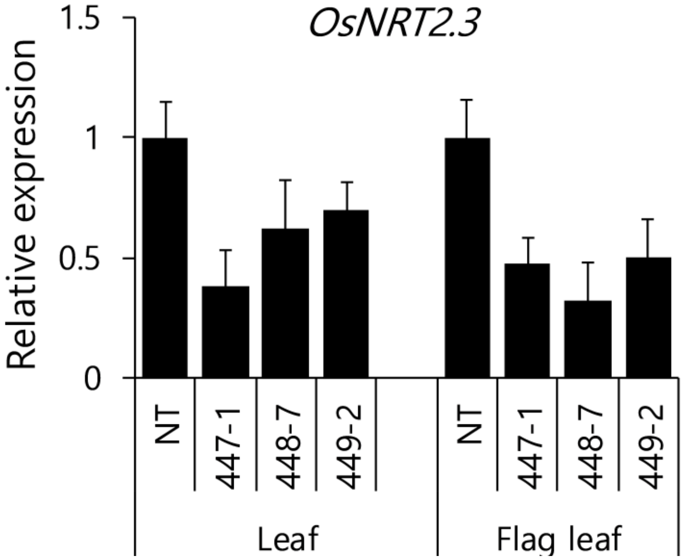

Figure S9. Expression of *OsNRT2.3* in leaf tissues of 14 DAF *OsUPS1*^{OX} plants.

Values are the mean ± SD of three biological samples and three technical replicates. All values were relative to WT.

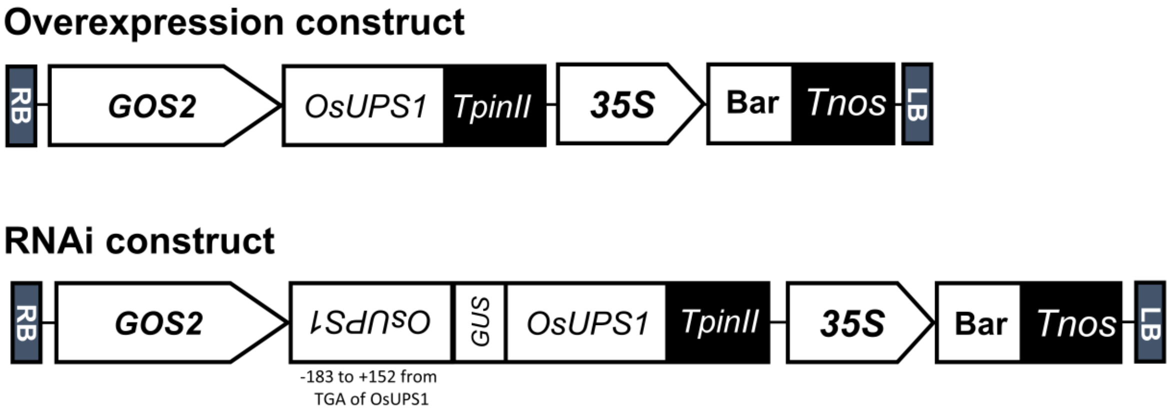

Figure S10. Vectors used for rice transformation with overexpression and silence cassettes. The expression cassettes were used to produce the overexpression $OsUPS1^{GOS2}$ and silencing $OsUPS1^{RNAi}$ lines. *Tnos*, the 3’ region of *nopaline synthase* gene; *TpinII*, 3’ region of the potato (*Solanum tuberosum*) *proteinase inhibitor II gene*; *Bar*, herbicide-resistant selection marker; 35S, 35S promoter of Cauliflower mosaic virus; LB, left border; RB, right border.

Table S1. Free amino acid content in leaf, flag leaf and panicles of 14 DAF plants.

	Leaf (µg/g)				Flag Leaf (µg/g)				Panicle (µg/g)			
	WT	±	*OsUPS1* ^{OX}	±	WT	±	*OsUPS1* ^{OX}	±	WT	±	*OsUPS1* ^{OX}	±
Aspartic acid	66.7	5.0	64.1	9.9	74.3	10.6	76.4	7.8	318.6	2.3	321.0	27.9
Glutamic acid	413.4	50.5	477.5	28.6	517.6	56.0	619.4	22.7	648.9	13.7	680.9	11.3
Asparagine	3.6	0.7	4.3	0.4	5.2	2.1	6.4	1.2	62.0	7.9	1054.9 *	88.4
Serine	56.3	3.4	72.2	5.0	80.2	7.5	101.5	13.8	174.7	16.4	283.08 *	26.5
Glutamine	65.5	4.2	61.0	5.9	86.5	8.4	84.8	16.7	413.3	13.3	932.4 *	103.0
Histidine	5.8	1.3	4.1	0.4	6.2	1.5	5.6	0.8	14.1	1.3	24.8	4.6
Glycine	21.1	1.9	37.3	7.9	31.3	2.8	54.8	10.0	20.1	3.0	56.4	14.8
Threonine	26.4	1.7	23.8	1.0	37.5	2.9	38.3	0.7	50.1	1.9	74.3	8.0
Arginine	4.8	0.9	4.8	0.2	4.3	1.3	6.1	0.8	39.6	2.5	165.7	61.3
Alanine	74.3	28.6	71.8	20.5	89.6	15.2	94.7	34.7	152.4	5.2	159.6	19.4
GABA	12.9	6.1	9.7	1.1	13.3	4.8	12.4	3.0	27.5	5.5	50.9	0.3
Tyrosine	10.9	0.7	8.2	0.5	8.0	0.2	8.2	0.1	31.0	2.1	31.9	1.7
Valine	43.8	16.2	39.5	11.9	53.7	14.5	55.7	10.2	61.9	22.3	79.7	18.0
Methionine	0.4	0.1	0.5	0.1	0.5	0.1	0.7	0.1	2.2	0.1	3.3 *	0.3
Tryptophane	6.7	0.7	6.2	0.2	6.8	0.6	7.3	0.3	14.1	0.4	14.9	1.7
Phenylalanine	20.4	2.4	14.8	0.3	16.7	0.3	18.0	0.9	11.4	2.1	13.0	1.7
Isoleucine	15.8	1.6	13.5	0.8	16.1	0.6	19.6	1.1	18.1	1.4	26.3 *	2.0
Leucine	16.7	2.4	14.3	0.9	11.8	0.9	16.5 *	0.4	15.3	1.7	21.0	1.9
Lysine	7.1	1.8	6.4	1.5	7.4	2.4	8.2	3.1	20.9	3.0	47.7	9.5
Proline	12.3	3.3	11.8	0.4	20.6	1.3	23.9	0.8	38.9	6.3	53.9	11.3
TOTAL AA	884.9	122.5	945.9	36.4	1087.6	112.3	1258.5	23.4	2135.3	66.0	4095.4 *	97.9

Asterisk represent significant differences between *OsUPS1* ^{OX} with WT by the Student's *t*-test (*, $P < 0.05$).

Table S2. List of oligomers used in this study.

Genotyping	Forward sequence	Reverse sequence
Gene	CAGCTCTAGGATTGCTAAAG	GACACATGCTGGCTGGAGTAG
T-DNA	TTGGGGTTTCTACAGGACGTAA	
qRT-PCR Target	Forward sequence	Reverse sequence
OsUPS1 (Os12g0503000)	GCCTGCCTTGGATCTCTTGT	GGAGGTGCTTGGTGAGTTCT
OsUPS2 (Os12g0502800)	GGACCATGTCCTTGGTTGTG	CACCCCCTTTGTCCTCTATG
OsUPS3 (Os12g0503300)	CAAGGATGAGGAGGAGAAGC	TATCGAAATCGTCGTCTTCGC
NRT2.3 (Os01g0704100)	ACGGAGACCGGGATCAAGTA	TCCCGGCTGTAGTACTCCTC
Atase1 (Os01g0873100)	TTTGGGGCTTGGTGGCAATA	CATCACCATGATGGCATTGGC
Atase2 (Os01g0873200)	GCGTGTGGCGATCGTATTTC	TTTCGCTGGGATGAACTGGG
Atase3 (Os05g0430800)	GGCTCCTCTGCCTTTGTAGG	TCTCGACTCTCAAGCCACCA
GARS (Os05g0270800)	GCGTCCGTCGCTTCTACTG	AACACCACGAGCCTCTTCCT
GART (Os08g0500900)	CAGCATGCACGTAATGAGGC	TCACACAACAAGCGGTGAGT
FGAMS (Os01g0888500)	TCTCTAGTCAGCGAGCCCTT	CCAACTGTCTGCTGTTGTGC
AIRS (Os03g0831500)	TCATCCGACAGGCAAACCAG	ATAGGTTCTGCCGTTTGGGG
AIRC (Os01g0199900)	GCTCGAGGAACTAGGTTGGG	GCCTCCACATGGACCATATCT
ASL (Os03g0313600)	CCAATTTCTCGGATGCAGCG	ATTGGCTCTGCAAGGACCTC
ATIC (Os08g0206600)	CAGGAGTCAAACAAGCACTGA	CCATGTACACTGGGGTGCAA
ASS1 (Os03g0699300)	TAGGGGGACTTCAGGAAGCA	TATCCATTGGACAGCAGCGG
ASS2 (Os03g0174500)	CCCAACGCTATGTCGAGAGG	TCAAGGCCAAGTTAGGGCTG
IMPDH (Os03g0780500)	TCCGTTTACCATCGCAACGA	GCCGAATTCGCTACAACAGG
GMPS1 (Os08g0326600)	GCAACAATGTGCGAGGTGTT	AAAAGTAGGCCACTCCACGG
XDH (Os03g0429800)	CCTGTCGGTGTTGGAACTCA	GGTGTCGGTGTGTCATCAGT
Uricase (Os01g0865100)	CCTCATTCCCACGGGTTCAA	TTCGAACCCGGACTGAGTTG
ALN (Os04g0680400)	GGACTACGGTGACGCGGTTA	CAGCTGCTGCTCTTGTACCA
AAH (Os06g0665500)	CAACTTTTCTGGGAAGCGCC	GAGCATTAGCAGTGCCCTCA
UGAH (Os07g0495000)	CAGCCCTACCACCAAAAGGT	GGCATAAGAGTCCACCAGCA
UAH (Os12g0597500)	ACGTGCAAGCTCGAAGGTAT	AGTTGCAACCGCTCCTAGTC
OsUbi1 (Os06g0681400)	ATGGAGCTGCTGCTGTTCTA	TTCTTCCATGCTGCTCTACC
